# Thermal Hofstadter Butterflies

**DOI:** 10.1021/acs.nanolett.6c01000

**Published:** 2026-07-14

**Authors:** Natalia Cortés, Bastian Castorene, Francisco J. Peña, Damian Melo, Sergio E. Ulloa, Patricio Vargas

**Affiliations:** † Departamento de Física, 28090 Universidad Técnica Federico Santa María, 2390123 Valparaíso, Chile; ‡ Instituto de Física, Pontificia Universidad Católica de Valparaíso, Casilla 4950, 2373223 Valparaíso, Chile; ¶ Facultad de Ingeniería, Universidad San Sebastián, Lago Panguipulli 1390, 5501842 Puerto Montt, Chile; § Department of Physics and Astronomy and Nanoscale and Quantum Phenomena Institute, 1354 Ohio University, Athens, Ohio 45701, United States; ∥ Departamento de Física, Universidad Técnica Federico Santa María, 2390123 Valparaíso, Chile

**Keywords:** magnetocaloric, fractal, Hofstadter, 2D lattice, thermodynamics

## Abstract

Fractal electronic spectra arising from the competition between lattice periodicity and magnetic flux are a fundamental hallmark of two-dimensional quantum systems. While the spectral properties of Hofstadter butterflies are well-documented, their thermodynamic response has remained remarkably unexplored. We present an original characterization of the electronic entropy *S*_*e*_ and specific heat *C*_*e*_, at half-filling, for square, honeycomb, and triangular lattices under a magnetic field. We demonstrate that these observables exhibit fast and slow magneto-thermo oscillations and pronounced magnetocaloric effects. We identify striking self-similarity in *S*_*e*_ and *C*_*e*_, tracing heart-shaped specific heat and tunnel-like entropy contours that repeat at specific lattice-dependent magnetic fluxes. Entropy minima at low temperatures act as fingerprints for the butterfly spines, resolving the underlying fractal spectra. These findings may establish thermal measurements as high-resolution spectroscopic probes, providing a robust framework for recognizing fractal signatures through thermodynamics in diverse nanostructures.

## Graphical abstract

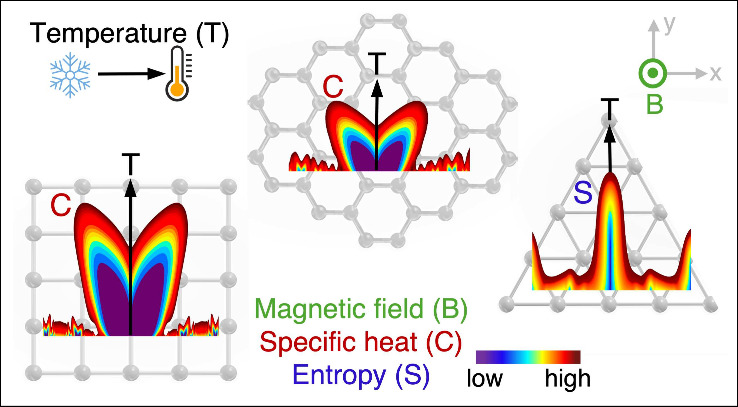

In 1976, Douglas Hofstadter published his seminal work exploring the spectral properties of noninteracting electrons confined to a two-dimensional (2D) square lattice subjected to a uniform magnetic field.^1^ This model has become a cornerstone study in a wide variety of systems, with lattice periodicities ranging from nanometers in quantum materials to centimeters in other setups. This special spectral system has motivated explorations in a host of modern condensed matter systems, resulting in new insights on quantum Hall phases, moiré heterostructures, ultracold Fermion gases, and topological materials. The model has inspired research on Chern number classifications and quantum Hall physics,^2^ to recent observations in moiré superlattices,^3–8^ engineered cold-atom systems in optical lattices,^9–11^ microwave cavities,^12,13^ two-dimensional photonic systems,^14^ and acoustic crystals.^15^

Hofstadter’s work centers on the importance of the energy spectrum of the system depending sensitively on whether the magnetic flux per lattice unit cell α is a rational or irrational number. For rational values of α, the system obeys Bloch’s theorem and displays an energy level structure with an integer number of magnetic subbands, such that the whole spectrum exhibits a beautiful fractal structure, famously known as the *Hofstadter butterfly*. This intricate spectrum, obtained by numerically solving for energy eigenvalues for rational values of α, illustrates the rich self-similar pattern that encodes the interplay between lattice periodicity and magnetic translation symmetry. The Hofstadter butterfly exemplifies how concepts from number theory (e.g., Diophantine equations and continued fractions) naturally emerge in the spectral analysis of quantum systems with appropriately commensurate parameters.

Despite extensive studies of its spectral properties and topological classification, the thermodynamic response of electrons of the Hofstadter butterfly’s fractal energy landscape has remained largely unexplored. Early work calculated magnetization and specific heat at low temperatures in the square lattice, revealing the characteristic gap structure through oscillations in these observables.^16,17^ How lattice geometry shapes thermodynamic response, however, has not been systematically addressed.

This work provides the first theoretical characterization of Hofstadter butterflies under temperature in square, honeycomb, and triangular geometries, bridging the gap between abstract fractal energy spectra and measurable thermodynamic quantities, and contributing to exploring further physical observables in these 2D lattice geometries.

Through the use of single-orbital tight-binding equations and Fermi–Dirac statistics, we perform a detailed analysis of the thermal behavior for electrons as a function of magnetic flux α and temperature *T* for the three lattice geometries across different parameter regimes. We present results for the electronic specific heat *C*_*e*_, and electronic entropy *S*_*e*_. We find that the thermodynamic response reflects universal thermal effects for the three lattices, as well as unique features at particular α flux values for each lattice geometry. Both thermal quantities reveal fractal signatures in each spectrum through intricate patterns in the α and *T* plane, highlighting phenomena such as fast and slow magneto-thermo oscillations and large magnetocaloric effects. These results may provide a clear roadmap for the experimental verification of the fractal nature of the spectra.

## Energy Spectra

The allowed eigenstates of an electron in a uniform magnetic field in a given lattice geometry are determined through single-band tight-binding models to capture the fine structure and recursive properties of the spectrum. The Hamiltonian *H* considers a uniform magnetic field **B** = *B ẑ* employing the Peierls substitution.^18^ In this framework, the hopping matrix elements acquire a phase factor due to the associated vector potential **A**, leading to the (spinless) Hamiltonian

1 $$H=-t\underset{\left\langle i,j\right\rangle }{\sum }e^{i\theta_{ij}}c_{i}^{†}c_{j}$$

where the sum runs over all nearest-neighbor pairs ⟨*i*, *j*⟩ in the lattice, and *c*_*i*_^†^ and *c*_*j*_ correspond to Fermionic operators creating and annihilating an electron at site *i* and *j*, respectively. The hopping amplitude *t* between nearest neighbors will be used as the unit of energy throughout the paper. The Peierls phase θ_*ij*_ is given by

2 $$\theta_{ij}=\frac{2\pi }{\Phi_{0}}\int_{r_{i}}^{r_{j}}A\cdot dr$$

with Φ_0_ = *h*/*e* the magnetic flux quantum, *h* the Planck constant, and *e* the elementary charge. For each geometry in Figure 1, *d* **r** parametrizes the path between two lattice sites, **r**_*i*_ and **r**_*j*_.

**Figure 1:**
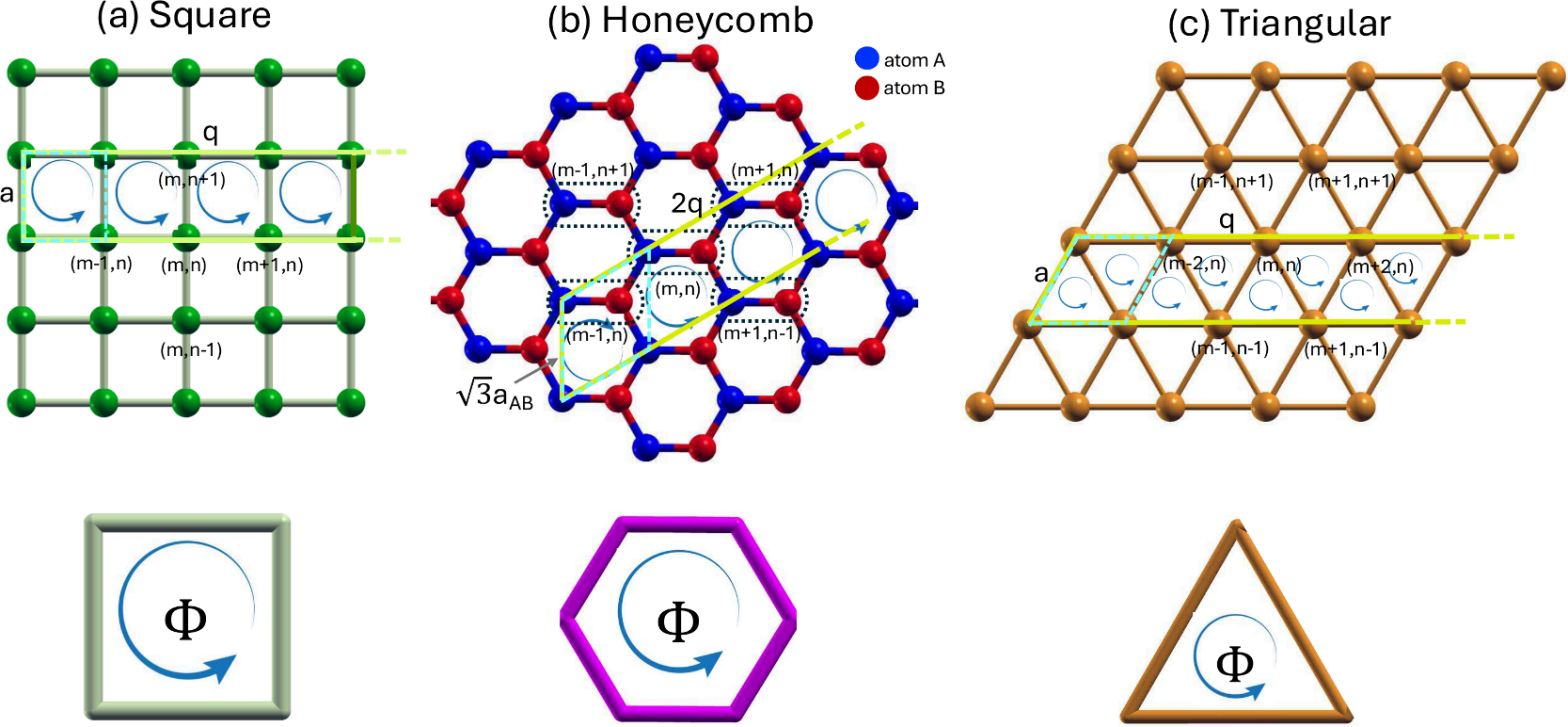
Top panels: (a) square, (b) honeycomb, and (c) triangular geometrical lattices. The light green rectangle in (a) and parallelograms in (b) and (c) show the magnetic unit cells, the cyan square in (a) and parallelograms in (b) and (c) represent the geometrical unit cells, *a*_AB_ is the distance between A-B atoms in b. Ordered pairs label the nearest neighbor sites to the (*m*, *n*) site in each lattice. Bottom panels show the corresponding elemental plaquettes with magnetic flux Φ = *α Φ*_0_, with rational α = *p*/*q*.

As we explicitly show in the Supporting Information (SI), solving the Schrödinger equation *Hψ* = *E ψ*, with *H* in eq 1, and *ε* = *E*/*t*, requires a rational dimensionless parameter α^1^

3 $$\alpha =\frac{\Phi }{\Phi_{0}}=\frac{p}{q}$$

where Φ is the magnetic flux through a single lattice plaquette (see Figure 1), and *p* and *q* are relative prime integers. This parameter provides essential details in the problem: it enumerates the magnetic subbands in each system, as *q* in the square and triangular lattices (and 2*q* in the honeycomb case); it also allows characterizing the symmetry of the energy spectrum under inversion about α for each lattice system.

Numerical diagonalization of the matrices associated with the eigenvalue equation for each lattice yields the eigenenergies {*ε*_*l*_(α)}, where *l* labels the states for each α value. These calculations yield the well-known symmetric spectra for the square and honeycomb lattices, while the triangular lattice yields an antisymmetric butterfly spectrum. The supplement contains the defining equations for the different lattice spectra needed to compute the thermodynamic response for each butterfly.

## Butterfly Thermodynamics

The three geometries described above exhibit different thermodynamic properties that depend strongly on the flux α and temperature *T*. We obtain the electronic internal energy *U*_*e*_ (not shown), specific heat *C*_*e*_, and entropy *S*_*e*_, for different magnetic flux per plaquette α and temperature. For concreteness, we consider the half-filling condition $\nu =\frac{1}{2}$ for both symmetric and nonsymmetric butterflies (see SI for a different ν value).

To calculate thermodynamic quantities, it is convenient to define the density of states (DOS) by

4 $$D_{\alpha }\left(\varepsilon \right)=\overset{Q}{\underset{l=1}{\sum }}\delta \left(\varepsilon -\varepsilon_{l}\left(\alpha \right)\right)$$

where *l* runs from 1 to *Q* for each value of α, with *Q* = *q* for the square and triangular lattice, and *Q* = 2*q* for the honeycomb lattice. A crucial characteristic of the electronic spectra in the bipartite square and honeycomb lattices is that they exhibit reflection symmetry about *ε* = 0, ensuring perfect electron–hole symmetry in the DOS, *D*_α_(*ε*) = *D*_α_(−*ε*). On the contrary, *D*_α_(*ε*) ≠ *D*_α_(−*ε*) for the triangular lattice (see supplement). We should note, however, that the full spectrum is traceless, so that ∑_*l* = 1_^*Q*^ *ε*_*l*_ *=* 0 for each α value in all three lattices. These DOS symmetries are useful for setting the chemical potential μ in the systems.

### Chemical Potential

In a half-filled system, $\nu =\frac{1}{2}$ , only the lower half of the *Q* energy levels are occupied in the ground state. Using the DOS in eq 4, the chemical potential at finite *T* satisfies the condition

5 $$\left\langle N\right\rangle =\int D_{\alpha }\left(\varepsilon \right)f\left(\varepsilon ,\mu ,T\right)d\varepsilon =\overset{Q}{\underset{l=1}{\sum }}f\left(\varepsilon_{l}\left(\alpha \right),\mu ,T\right)=\nu Q$$

where *f*(*ε*,μ,*T*) = [1 + *e* ^(*ε*–μ)/*k*_*B*_ *T*^]^−1^ is the Fermi–Dirac distribution function, with *k*_*B*_ the Boltzmann constant. In the square and honeycomb lattices, the chemical potential μ = 0 satisfies the half-filling condition, eq 5, for all values of α and *T* due to the electron–hole symmetry of their spectra.

The corresponding occupation constraint defining μ = μ­(α, *T*) in the triangular lattice is given also by eq 5. Due to the spectral asymmetry of the triangular lattice butterfly, μ is seen to change its value with magnetic flux α, even at zero temperature, to satisfy the half-filling condition. This is illustrated in Figure 2, which shows sharp changes in μ whenever the spectrum has gaps. It is interesting to observe that μ­(α,*T*) in Figure 2 is antisymmetric with respect to α = 1/2, so that $\mu \left(\frac{1}{2}-\delta ,T\right)=$ $-\mu \left(\frac{1}{2}+\delta ,T\right)$ , with δ rational, and that this relation is valid for all temperatures.[Fn fn1] The strong variation of μ with α reflects the nonmonotonic DOS of the spectrum—a characteristic also present in the symmetric butterflies away from half-filling (see SI), but hidden here.

**Figure 2:**
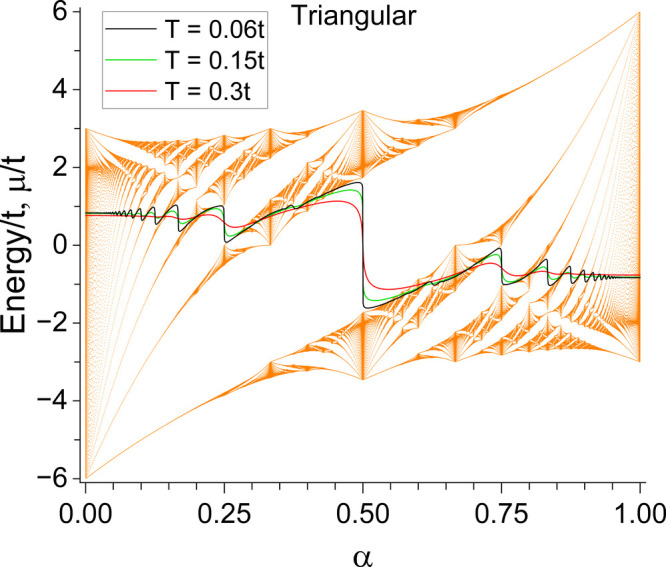
Chemical potential curves μ­(α,*T*) (solid lines) as a function of α = *p*/*q* (*q* = 1123) and different values of temperature *T* under the condition of half-filling in the triangular lattice; all energies in units of the hopping parameter *t*. The energy spectrum *ε* is shown behind the curves, where the sharp drops in μ are seen to occur within the gaps in the spectrum.

### Thermodynamic Quantities

The chemical potential μ = 0 for symmetric butterflies, and μ­(α,*T*) for the antisymmetric butterfly, all at half-filling, are used to calculate the electronic specific heat *C*_*e*_ and entropy *S*_*e*_ shown below. The electronic specific heat is calculated through *C*_*e*_ = ∂ *U*_*e*_/∂ *T*

$$C_{e}\left(\alpha ,T\right)=\frac{\partial }{\partial T}\int \varepsilon D_{\alpha }\left(\varepsilon \right)f\left(\varepsilon ,\mu ,T\right)d\varepsilon$$ 6

and the entropy in terms of the Fermi–Dirac function *f* is

7 $$S_{e}\left(\alpha ,T\right)=-\int D_{\alpha }\left(\varepsilon \right)\left[f\ln f+\left(1-f\right)\ln\left(1-f\right)\right]d\varepsilon$$

The DOS in eqs 6 and 7 is given by eq 4. The supplement contains detailed information for analytical and numerical thermodynamic quantities for all butterflies.

## Finite-Temperature Results

The thermodynamic calculations are performed for all rational flux values of α ∈ [0,1]­(eq 3), with *q* = 1123 and *p* integer ∈ [0,*q*]. Choosing a large prime number *q* ensures both computational tractability and sufficient resolution to capture the fractal structure of the three lattice butterflies, together with their thermodynamic response at all temperatures.[Fn fn2]

### *T*-α Thermodynamics

We display specific heat and entropy contour maps over the *T* and α plane for each of the three lattice geometries. These representations allow one to correlate the spectral characteristics, such as gaps, butterfly spines, and dense subband regions having high DOS with clear features of thermal excitations in the thermodynamic functions, especially at low temperatures where fractality is preserved.

**Square lattice.** Figure 3 presents results for the square lattice, the original butterfly. Both thermodynamic functions, *C*_*e*_ and *S*_*e*_ are symmetric about $\alpha =\frac{1}{2}$ , inheriting the symmetry of the energy spectrum (see supplement). At low temperatures (*T* ≲0.2*t*) these functions are most sensitive to the gap structure near half filling, μ = 0, exhibiting intricate fast magneto-thermo oscillations coincident with the spectral features. Constant specific heat curves (some indicated by light-yellow lines) in panel [Fig fig3]a appear as heart-like shapes of different sizes, depending on the corresponding nearby spectral gaps; the largest ‘heart’ is centered at $\alpha =\frac{1}{2}$ , and replicas centered at the butterfly spectral ‘spines’ (for values $\alpha =\frac{1}{2},\frac{1}{4},...$ ). The *C*_*e*_ hearts indicate deep, broad minima (dark brown zones) that connect with sharp low-*T* oscillations as α varies. The suppressed *C*_*e*_ inside the hearts is associated with low DOS. As temperature increases, thermal excitations overcome the spectral gaps, broadening out fine-scale structures and leading to weaker isothermal variations, and a *C*_*e*_ maximum near $\alpha =\frac{1}{2}$ .

**Figure 3:**
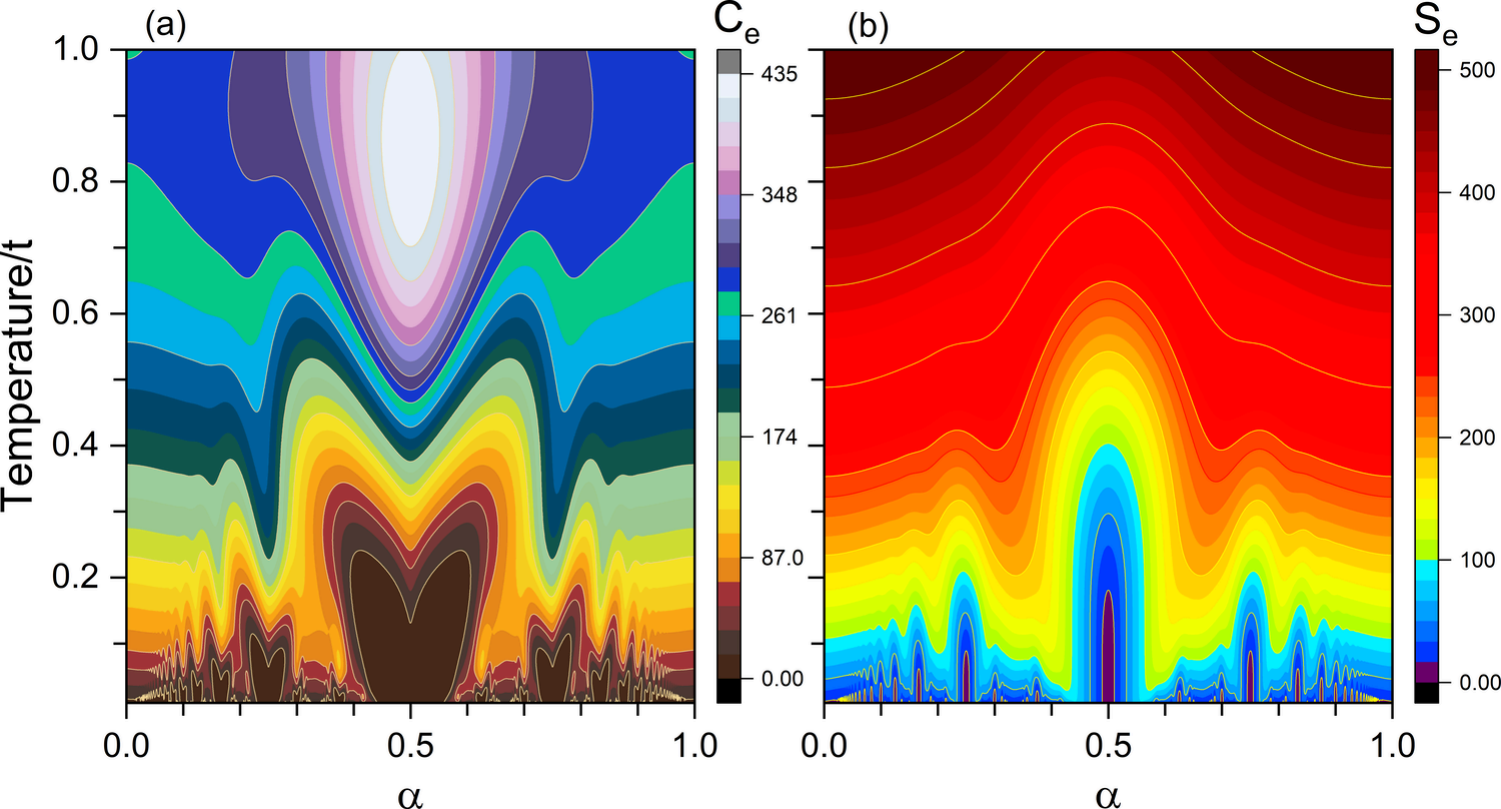
**Square lattice**. Contour plots for (a) electronic specific heat *C*_*e*_(α, *T*), and (b) entropy *S*_*e*_(α, *T*), as a function of the magnetic flux parameter α = *p*/*q* (*q* = 1123), and temperature *T*/*t*. Solid light-yellow lines represent selected constant-*C*_*e*_ or constant-*S*_*e*_ contours. Color bars in units of *k*_*B*_. Notice rich oscillatory structure associated with spectrum at extreme α values.

The entropy for the square lattice in [Fig fig3]b shows deep minima with ‘tunnel’-like shapes near spectral gaps (purple zones), also centered at the butterfly ‘spines’. At α = 1/2, the vanishing DOS near half filling results in slow entropy growth as *T* increases (see SI for *S*_*e*_ vs *T* curves). The deep entropy minimum up to *T* ≈ 0.2*t* shows the restricted phase space for thermal excitations. Constant-temperature entropy curves show sharp minima at the butterfly spines, especially clear at low *T*. The entropy is larger at higher temperatures, as expected, showing less oscillatory structure, a local maximum around α = 0,1, and becoming nearly featureless for *T* ≳ 1*t*.

**Honeycomb lattice.** Figure 4 shows results for the symmetric honeycomb butterfly (SI Figure 1b). The specific heat and entropy functions are symmetric about $\alpha =\frac{1}{2}$ , as expected. At low temperatures, constant-*C*_*e*_ curves in this case (light-yellow lines) have also heart-like shapes, centered at the butterfly spines, $\alpha =\frac{1}{2},\frac{1}{3},\frac{1}{4},...$ . Although the minima are not as broad as for the square lattice, given the overall smaller gaps in the spectrum near half filling, they show oscillations that coincide with the spectral structures (SI Figure 3b). Near fluxes α = 0 and 1, *C*_*e*_ is largely suppressed at low *T*, in contrast to the square lattice. This effect is directly connected to the different thermal activation processes for both lattices. See SI for *C*_*e*_ vs *T* cuts.

**Figure 4:**
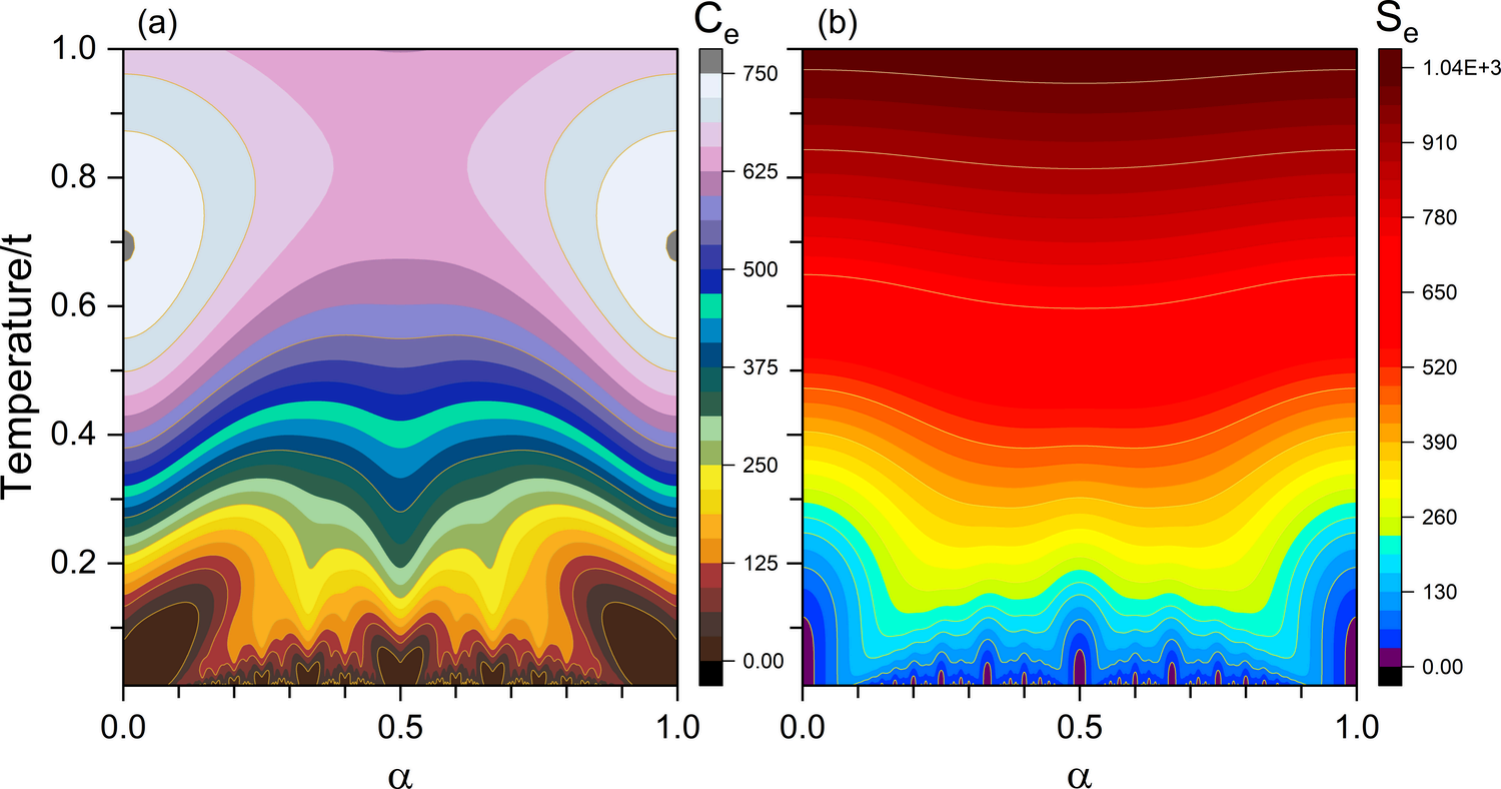
**Honeycomb lattice**. Contour plots for (a) electronic specific heat *C*_*e*_(α,*T*), and (b) entropy *S*_*e*_(α, *T*) as a function of the magnetic flux parameter α = *p*/*q* (*q* = 1123), and temperature *T*/*t*. Solid light-yellow lines represent selected constant-*C*_*e*_ or constant-*S*_*e*_ contours. Color bars in units of *k*_*B*_.

The isentropic contours in the honeycomb lattice in Figure 4b, also show tunnel-like shapes at low *T* (purple zones) with clear local peaks centered on the respective butterfly spines, and with sizes that indicate the overall density of the spectral honeycomb features (blue contours). As *T* increases, the isentropic curves lose their oscillatory behavior across α, and become nearly featureless at lower temperatures than in the square lattice.

**Triangular lattice.** Despite the lower symmetries in the spectrum of the triangular lattice seen in Figure 2, the thermodynamic functions such as *U*_*e*_, *C*_*e*_, or *S*_*e*_ recover inversion symmetry with respect to $\alpha =\frac{1}{2}$ at this filling factor.[Fn fn1]

It is interesting to see narrow and deformed heart-like shapes here, with nonsymmetric minima (except at $\alpha =\frac{1}{2}$ ), defined by the constant-*C*_*e*_ curves in Figure 5a, especially at low *T* (light-yellow lines). These minima appear whenever μ traverses a subband gap in the spectrum, for $\alpha =\frac{1}{2},\frac{1}{4},...$ , see Figure 2. The deformed hearts also delimit regions of low specific heat values (dark brown), flanked by high value regions (yellow), whenever μ is in a dense subband region. Similar to the square lattice, local minima of *C*_*e*_ evolve into local maxima at higher temperatures, reaching a hot spot at $\alpha =\frac{1}{2}$ near *T* ≃ 1*t*. Thermal excitations into dense spines in the triangular spectrum take over, making *C*_*e*_ contours smooth as *T* increases.

**Figure 5:**
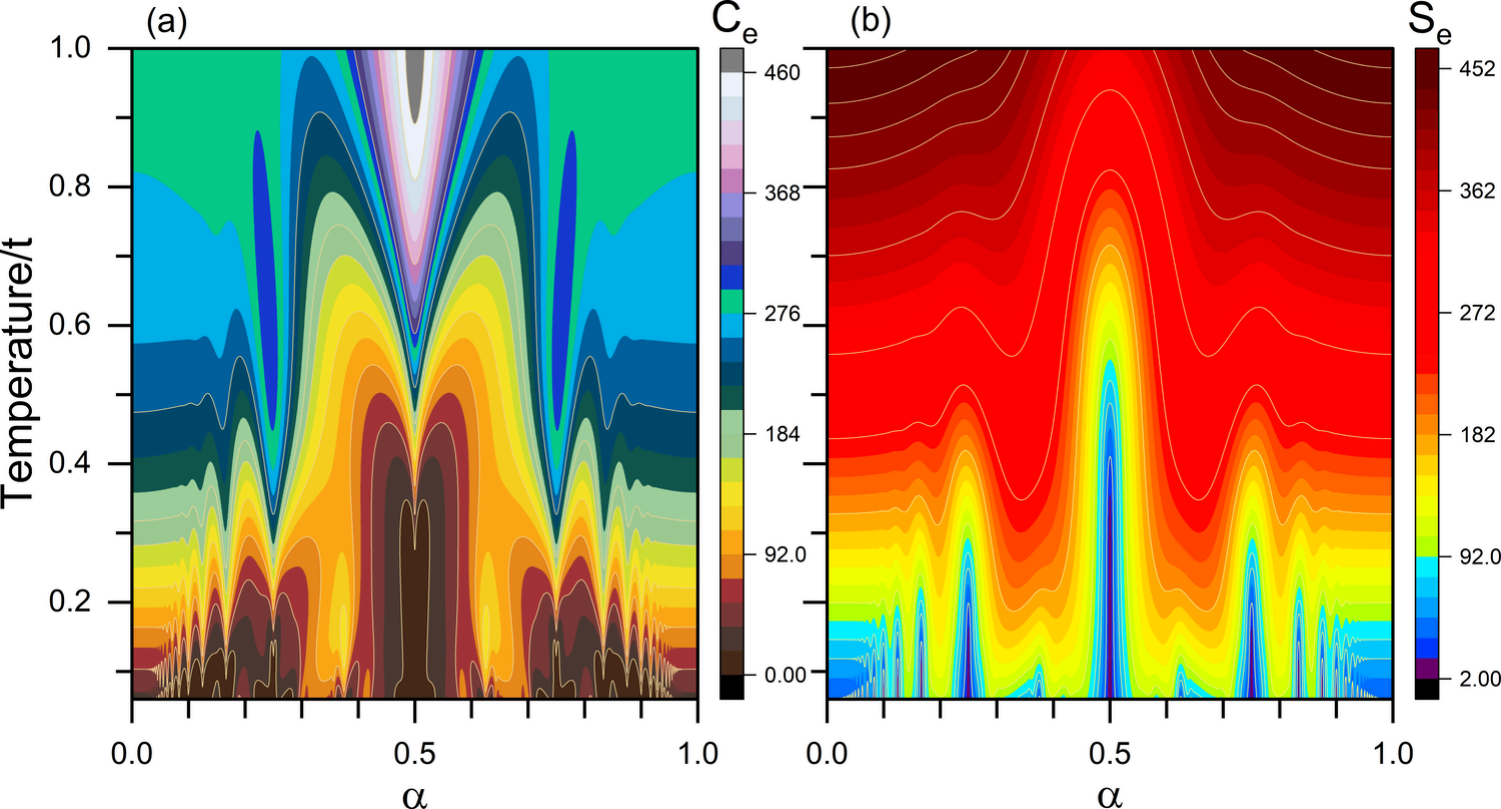
**Triangular lattice**. Contour plots for (a) electronic specific heat *C*_*e*_(α, *T*), and (b) entropy *S*_*e*_(α, *T*), as a function of the magnetic flux parameter α = *p*/*q* (*q* = 1123), and temperature *T*/*t*. Solid yellow lines represent selected contours in both panels. Color bars in units of *k*_*B*_.

The entropy in the triangular lattice, Figure 5b, shows deep minima within the ’tunnels’ (purple regions) whenever μ is in a gap, that clearly accompany the pattern of hearts seen in panel a. The largest and narrowest tunnel around $\alpha =\frac{1}{2}$ and vanishing specific heat come from the main spectral gap, generating a persistence of their structures as *T* raises, see supplement. This behavior may be linked to the reduced symmetry of the triangular lattice relative to the square and honeycomb lattices, as spectral structure varies in different Bravais lattices.^19^

### Entropy Oscillations and Magnetocaloric Effect

It is important to analyze the physical significance of the complex behavior of the isentropes in the different lattices in Figures 3, 4, and 5, panels b. The calculated electronic entropy *S*_*e*_(α, *T*) provides a map of the system’s magnetocaloric potential for cooling and/or heating as the magnetic field changes.^20–23^ By following the isentropes (light yellow lines), one can observe the temperature response of each lattice to variations in the magnetic flux α. The resulting variations in *T* required to maintain a constant *S*_*e*_ indicate that each system undergoes heating and cooling cycles as the flux is modulated. The strong entropy variations as *T* changes are associated with convergence of states with flux, such as in the spectral butterflies’ spines, producing alternating regions of high and low DOS near μ. All geometries show fast *S*_*e*_ oscillations. Notably, the steepest temperature gradients occur at low *T*, where the entropy is more strongly affected by the fine structure of the energy spectrum.

The square and triangular butterflies exhibit a larger magnetocaloric effect (MCE), centered at the spines $\alpha =\frac{1}{2},\frac{1}{4},...$ , where small flux changes are needed for an isentrope to be preserved, producing large variations in *T* (blue and yellow *S*_*e*_ gradients in the maps). Particularly, approaching $\alpha =\frac{1}{2}$ , the accumulation of isentropes toward minima suggests a high cooling efficiency near this flux value, see SI for some MCE numbers. The robust MCE could, in principle, have important applications in thermal engines that utilize these systems. As pointed out above, however, as *T* increases, the distinct quantum signatures are masked by thermal broadening, leading to an evolution from a highly oscillatory adiabatic response to a more monotonic regime and a less efficient MCE.

### Fractal Signatures in Different Lattices

Figure 6 displays isotherms for the electronic specific heat (*TC*_*e*_) and entropy (*TS*_*e*_) as a function of the magnetic flux parameter α at fixed low *T* for all three lattices. These curves are superimposed on a portion of the energy spectrum to facilitate the identification of the thermodynamic behavior with spectral fractal features of each lattice. As we consider half-filling in all cases, the chemical potential for the square and honeycomb lattices (a and b panels) is constant at μ­(α) = 0 (not shown), while for the triangular lattice in (c), μ­(α) is indicated by a black dashed line (that starts nonzero at α = 0, and after oscillations reaches $\mu \left(\alpha =\frac{1}{2}\right)=0$ , and changes sign beyond, as discussed above).

**Figure 6:**
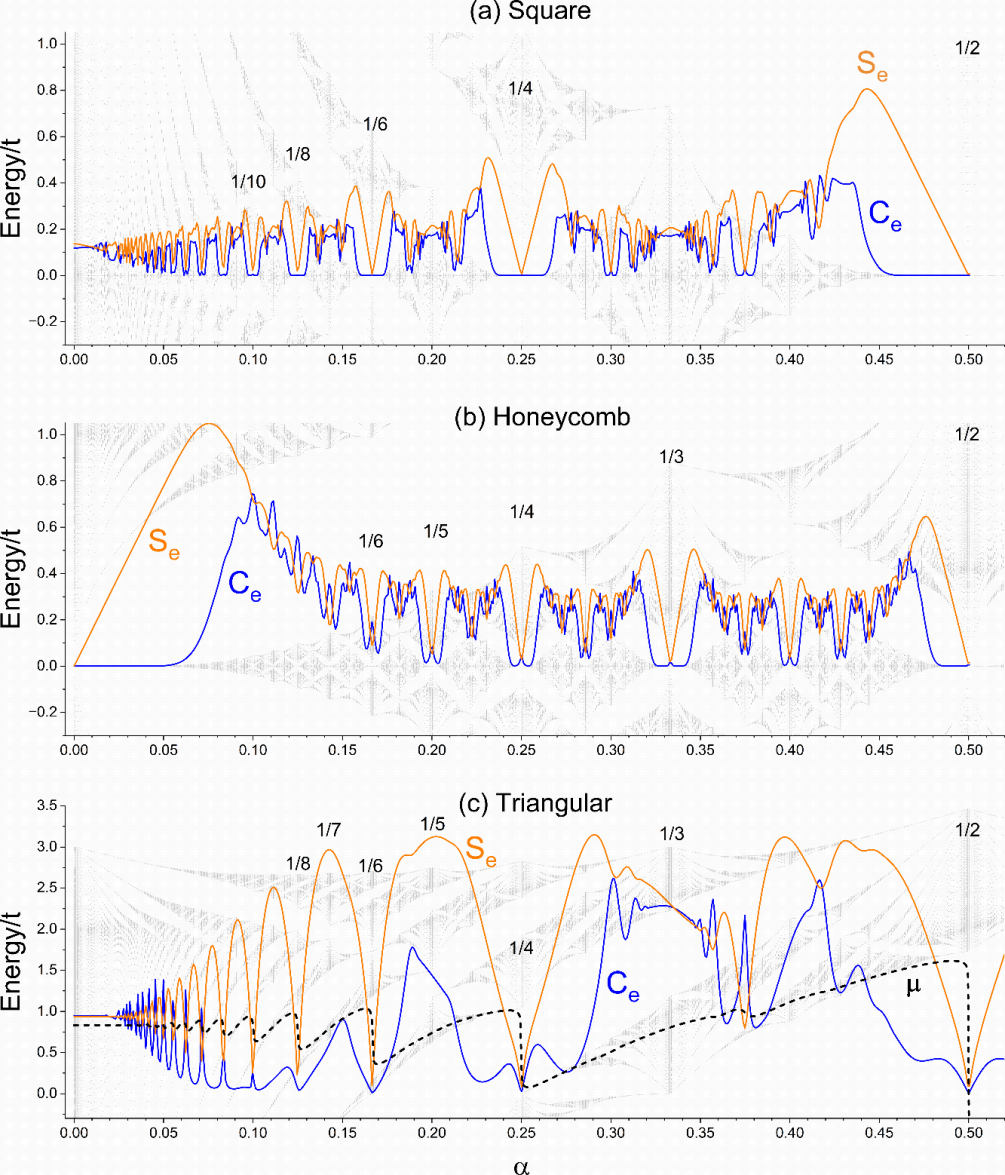
Electronic entropy *S*_*e*_ (orange lines) and specific heat *C*_*e*_ (blue lines) isotherms superimposed on a portion of the energy spectrum. (a) Square lattice, (b) honeycomb lattice, (c) triangular lattice; all at half-filling. Isotherms in (a)-(b) are for *T* = 0.01*t*, plots are *TC*_*e*_ and *TS*_*e*_; for (c) *T* = 0.06*t*, and shown are *TC*_*e*_/2, and *TS*_*e*_/2 to fit scale, and μ­(α) curve with a dashed-black line. *k*_*B*_ = 1. Note that minima in *S*_*e*_ occur for α values associated with the ‘spine’ of the butterflies, such as $\alpha =\frac{1}{4},\frac{1}{2},...$ . Differences seen among different lattices are closely related to their spectra near the Fermi level. See text for discussion.

It is interesting to notice a number of similarities and differences among the various lattice isotherms *S*_*e*_ and *C*_*e*_, all connected to features of the fractal spectrum given by the low temperature values used here (*T* = 0.01*t* in a, b; *T* = 0.06*t* in c). The thermal energy *k*_*B*_ *T* acts as a resolution limit in all cases. Since *k*_*B*_ *T* is much smaller than the primary gaps, the major features of the butterflies are well-resolved.

The thermal response is determined mainly by the DOS near the chemical potential μ. For small α, the square and triangular lattices have nearly constant *C*_*e*_ and *S*_*e*_ isotherms, as the DOS at μ is nonzero, while the honeycomb lattice shows vanishing *C*_*e*_, but linear *S*_*e*_. Similar behavior is seen with minima located at α values that connect with the butterfly spines, produced by the vanishing DOS near μ as the subbands asymptotically ‘touch’ each other. The pattern of vanishing specific heat and linear entropy is clearly repeated for the square and honeycomb lattices, whenever μ is near a minimal DOS. The low DOS negates (or suppresses) thermal excitations at low temperatures, which changes as α shifts to higher DOS regions in the spectrum.

The minima for *S*_*e*_ at the butterfly ‘spines’ represent a clear signature of fractality, evident for $\alpha =\frac{1}{8},\frac{1}{6},\frac{1}{4},\frac{1}{2}$ in panel a; and $\alpha =\frac{1}{4},\frac{1}{3},\frac{1}{2}$ in panel b. The magnetic flux patterns in the square lattice at $\alpha =\frac{1}{2n}$ , and $\alpha =\frac{1}{n}$ in the honeycomb lattice (with integer *n* > 0), allow one to ‘observe’ the underlying butterfly spectrum by looking for entropy minima.

It is also interesting to notice oscillations in *C*_*e*_ and *S*_*e*_ whenever μ traverses a dense subband region in the spectrum. These can be seen in the square lattice for α ≃ 0.2, 0.285, 0.33, 0.4, and repeated at even smaller α values in a self-similar pattern. In the honeycomb lattice, similar oscillations appear in paired structures near flux α ≃ 0.18, 0.22, 0.285, 0.375,···, smoothly evolving into featureless curves for smaller α.

The triangular lattice is rather different, as the chemical potential traverses large gaps as the flux is changed to maintain half-filling. The total vanishing of the DOS midgap produces sharp minima in both *C*_*e*_ and *S*_*e*_, without the nearly flat plateaus seen in *C*_*e*_ in panels a and b. For smaller α, with smaller spectral gaps, the sharp minima in *C*_*e*_ transition to peaks that coincide with *S*_*e*_ minima, showing complementary oscillations for both thermal quantities. This is clearly seen for $\alpha \leq \frac{1}{10}$ . Notice that panel c shows a higher *T* isotherm (six times higher than in panels a and b), which smooths out many features. However, the self-similar oscillations that occur whenever μ traverses a dense subband region are also visible (here near $\alpha =\frac{1}{3},\frac{1}{5}$ ). The lower symmetry of the triangular lattice spectrum and the varying μ at half-filling result in a more complex behavior in the thermodynamic quantities, as the butterfly patterns are compressed and stretched in nonsymmetric fashion, when compared to those in the square and honeycomb lattices.

All of these results demonstrate that even at finite, but low, temperatures, the electronic entropy and specific heat serve as sensitive probes of the Hofstadter butterfly spectral patterns. The close correspondence of thermodynamic minima with butterfly spines and spectral gaps, as well as regions of dense subband levels, provides evidence that *S*_*e*_ and *C*_*e*_ encode the fractal structure of the spectrum in all of these lattices.

We also note that there is a close relationship between entropy and quantum capacitance, with the latter likewise revealing the butterfly’s fractal spectral features, especially at low temperatures, as we discuss in detail in the SI.

The thermodynamic responses captured by the electronic specific heat *C*_*e*_ and entropy *S*_*e*_ provide insight into each butterfly spectrum, seen as distinctive oscillations and repeated patterns at special magnetic flux values α that depend on the lattice geometry. At low and moderate temperatures these patterns in the *T*-α contour maps show ‘heart’-like shapes for the specific heat, and ‘tunnel’-like isentropic shapes of diverse sizes and widths. The largest *C*_*e*_ hearts are symmetric and centered at $\alpha =\frac{1}{2}$ or 1, with deep minima inside them in all lattices. However, the heart symmetry is broken for $\alpha \neq \frac{1}{2}$ in the triangular lattice due to the asymmetric spectra and varying chemical potential at half filling. The tunnel-like structures in *S*_*e*_ contours are symmetric as α varies, with isentropes converging near mid flux for the three lattices, indicating a high cooling efficiency with only small flux variations. The honeycomb lattice, in particular, exhibits large isentropic changes near α = 0 and α = 1, suggesting its utility in adiabatic cooling cycles at accessible fluxes. All these results reveal a robust magnetocaloric potential inherent in these fractal electronic systems.

The low-temperature behavior under variations of α can serve as an alternative window into the fractal nature of the spectra. Specifically, entropy minima provide a clear fingerprint for the butterfly spines at characteristic flux α = 1/2,1/4,1/6,··· for the square lattice, and α = 1/2,1/3,1/4,··· for the honeycomb lattice. Our calculations may help identify repetition patterns where thermal fractal signatures emerge, and assist in comparison with experiments.

Looking ahead, recent predictions of Hofstadter butterflies in 2D covalent organic frameworks (COFs) point to promising material platforms with intrinsically large lattice constants,^24^ allowing high-α regimes to be reached with experimentally accessible magnetic fields, opening an experimental scenario for the observation of Hofstadter butterflies in 2D systems. Other large-scale covalently linked organic structures (CLOS) may also allow studies at accessible magnetic fields.^25^ The wide availability of CLOS and COFs with large lattice constants may offer interesting possibilities to explore some of our predictions.

While isolating the electronic contribution from the lattice background in a 2D material is experimentally challenging, this has recently been achieved in graphene,^26^ and a recent report on quantized heat flow in a Hofstadter butterfly system is exciting.^27^ We note that the moiré period in bilayer graphene is ≃ 10 nm, with a bandwidth of ≃10–30 meV, which yields *t* ≃ 5 meV ≃ 60 K as energy scale. The magnetic field for $\alpha ≃\frac{1}{2}$ is then *B* ≃ 20 T, well-within the reach of modern experiments. These scales would yield sizable MCE of ≃ 0.2*t*, corresponding to a few kelvin.

Measurements of specific heat and entropy in modern 2D materials may provide direct signatures of fractal electronic spectra, establishing thermodynamic observables as high-resolution spectroscopic probes of fractal states that complement transport, capacitance, and local spectroscopic measurements.^3,6,8^ Finally, we note that the possible quantization of other thermal quantities, such as the Seebeck coefficient,^28,29^ and their relation to topological invariants in these spectra is a fascinating open question, especially as strong electronic correlations may be at play.^30^

## Supplementary Material



## Footnotes

1 This condition is connected to the antisymmetry of the spectrum, $-\varepsilon_{l}\left(\frac{1}{2}+\delta \right)=\varepsilon_{q-l}\left(\frac{1}{2}-\delta \right)$ , which together with its null trace, ∑_*l*_ *ε*_*l*_ = 0 ∀α, results in symmetric thermodynamic functions, such as $C_{e}\left(\frac{1}{2}+\delta \right)=C_{e}\left(\frac{1}{2}-\delta \right)$ , as seen in the results shown.

2 To capture the rich *T* dependence of thermodynamic functions, we employ an exponential mesh, *T*_*k*_ = *T*_1_(T_2_/*T*_1_)^(*k*−1) /(*N*_*p*_−1)^, with *k* = 1,···,*N*_*p*_. Typically, *N*_*p*_ = 200, *T*_1_ = 0.01*t* and *T*_2_ = 1*t*.

## Notes

Equations defining spectra and spectral plots for different lattices. Contour maps and α cuts of *C*_*e*_ and *S*_*e*_ vs *T*. DOS and ν = 0.6 results. Magnetocaloric effect numbers and quantum capacitance (PDF)
