# Supplementary material for: Thermal Hofstadter Butterflies
Source: Nano Lett. 2026 Jul 14;26(30):9743–50. doi: 10.1021/acs.nanolett.6c01000 (PMC13484923; doi:10.1021/acs.nanolett.6c01000)
Supplement: Supplementary file 1 [file nl6c01000_si_001.pdf]

# Thermal Hofstadter Butterflies
# Supporting Information

Natalia Cortés,*,† Bastian Castorene,‡,∥ Francisco J. Peña,¶ Damian Melo,‡,∥ Sergio E. Ulloa,*,§ and Patricio Vargas†

†*Departamento de Física, Universidad Técnica Federico Santa María, 2390123 Valparaíso, Chile*

‡*Instituto de Física, Pontificia Universidad Católica de Valparaíso, Casilla 4950, 2373223 Valparaíso, Chile*

¶*Facultad de Ingeniería, Universidad San Sebastián, Lago Panguipulli 1390, Puerto Montt, Chile*

§*Department of Physics and Astronomy and Nanoscale and Quantum Phenomena Institute, Ohio University, Athens, Ohio 45701, USA*

∥*Departamento de Física, Universidad Técnica Federico Santa María, 2390123 Valparaíso, Chile*

E-mail: natalia.cortesm@usm.cl; ulloa@ohio.edu

## Symmetric Butterflies

The presence of magnetic field **B** breaks the discrete translational symmetry of electrons in 2D lattices (in addition to time-reversal symmetry), due to the associated vector potential **A**. One can then construct a magnetic supercell, as shown in Fig. 1 in the text, that recovers translational invariance for an appropriate choice of gauge (see below). These choices lead to magnetic phases in the Hamiltonian that produce energy spectra with reflection symmetry

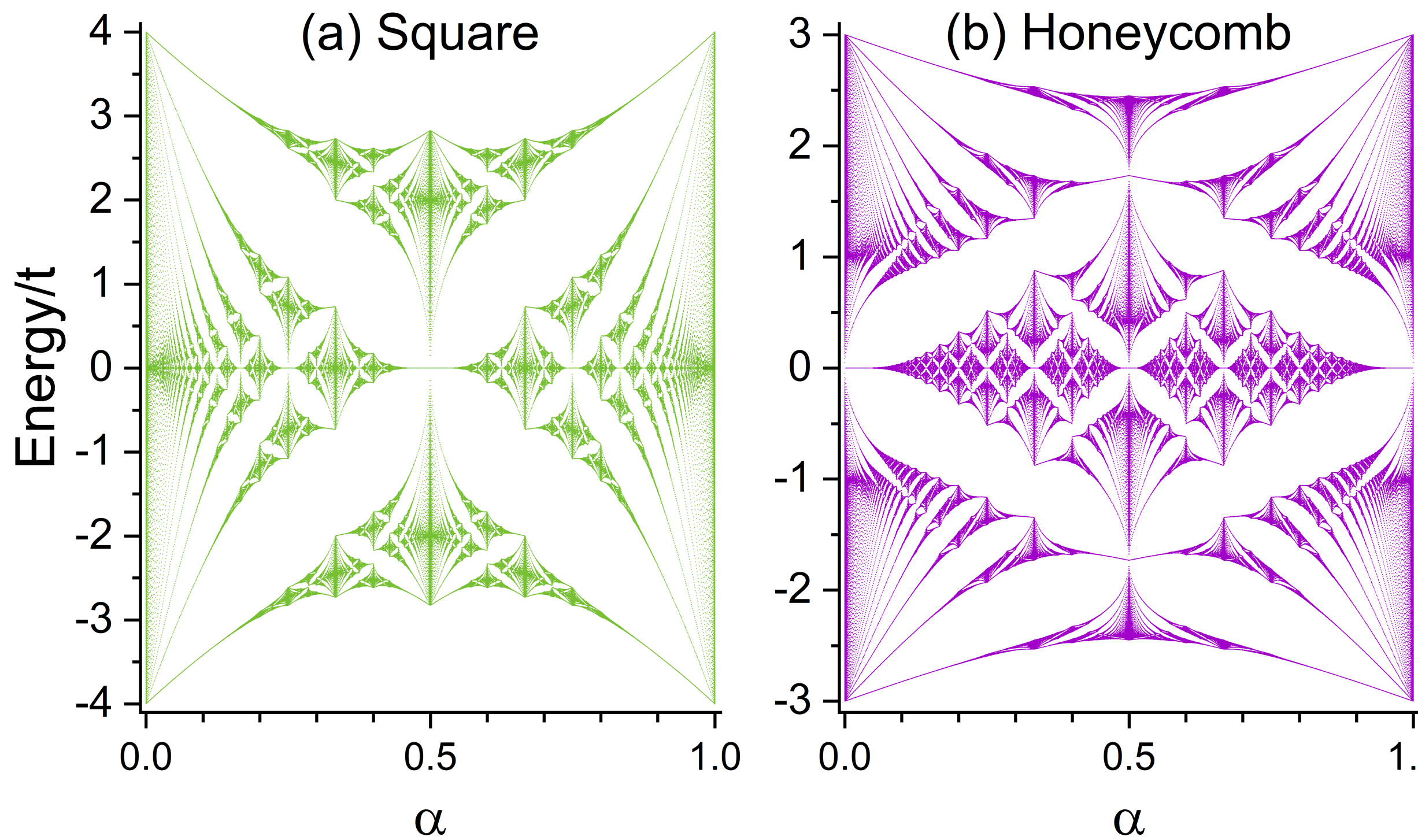


Figure 1: Electronic energy spectra in units of the hopping parameter $t$ as a function of $\alpha = p/q$, for (a) square lattice, and (b) honeycomb lattice. For these spectra $\alpha = p/q$, $p = 0, 1, .., q$, and $q = 1123$.

with respect to both zero energy and $\alpha = 1/2$.[1,2] The symmetry about $\alpha = 1/2$ for both square and honeycomb lattices in their respective butterfly spectrum is understood from Eq. 3 of the main text. One can *normalize* the lattice geometries by considering the magnetic flux per square and hexagonal plaquette constant equal to $\Phi/\Phi_0 = \alpha$. In doing that, $\alpha$ has a period of 1 for both symmetric lattices, as clearly seen in Fig. 1.

### Square lattice

It is convenient to use the Landau gauge for the vector potential $\mathbf{A} = (0, Bx, 0) = Bx\hat{y}$. The Hamiltonian keeps translational symmetry along the $y$-direction and the phases in Eq. 2 in the text only appear for hops along the $y$ direction. The wavefunction at each site $\mathbf{R}_{mn} = ma\hat{x} + na\hat{y}$ (with lattice constant $a$) is then given by $\Psi(m, n) = \psi(m)\, e^{ik_y na}$, where

$k_y$ is the conserved momentum, and the tight-binding equation reads[1]

$$\psi(m-1) + 2\cos(2\pi m\alpha - k_y)\psi(m) + \psi(m+1) = \varepsilon\psi(m), \qquad (1)$$

with energy eigenvalues $\varepsilon = E/t$. The magnetic flux per square plaquette in this geometry is $\Phi = BS = Ba^2$, and we have set $a = 1$ in Eq. 1. Notice that Eq. 1 is invariant under the change $m \to m + q$, for rational values of $\alpha$. This guarantees $\psi$ to fulfill a periodicity condition $\psi(m) = \psi(m+q)$, and defines a magnetic supercell of length $q$ along the $x$ direction.

**Honeycomb lattice**

The tight-binding model for the honeycomb lattice includes sublattices A and B in the hexagonal unit cell, Fig. 1b in the text. Using the vector potential $\mathbf{A} = Bx\hat{y}$ leads to different phases along particular hoppings in the hexagonal geometry.[3] Using these phases, and the conservation of $k_y$ momentum, one can decouple the resulting tight-binding equations for the two sublattices, and obtain separate relations for each of them. For example, for sublattice B, $\Psi^B(\mathbf{R}_{mn}) = e^{ik_y y_{mn}}\,\psi(m)$, one can write

$$e^{ik_y/2}u_m\psi(m-1) + u_{m+1}v_{m+1}\psi(m) + e^{-ik_y/2}u_{m+1}\psi(m+1) = (\varepsilon^2 - 1)\psi(m), \quad (2)$$

with coefficients

$$u_m = 2\cos\Big(\frac{k_y}{2} - \alpha\pi(m - \frac{1}{6})\Big),$$
$$v_m = 2\cos\Big(\frac{k_y}{2} + \alpha\pi(m - \frac{1}{6})\Big). \qquad (3)$$

The index $m$ labels the position of a single A-B dimer at $\mathbf{R}_{mn} = m\mathbf{a}_1 + n\mathbf{a}_2$, with $\mathbf{a}_1 = a(\sqrt{3}, 1)/2$, $\mathbf{a}_2 = a(0, 1)$, and the honeycomb lattice constant $a$ has been set to 1 in Eq. 2. The magnetic flux in this geometry is $\Phi = BS$, with $S = \sqrt{3}a^2/2$ the area of the hexagonal plaquette, Fig. 1b. Notice that the magnetic supercell of period $2q$ is implicit

in the coefficients, with $u_m = u_{m+2q}$, and $v_m = v_{m+2q}$, and the wave functions fulfill the condition $\psi(m) = \psi(m+2q)$.

The coefficients in Eqs. 1 and 2 are unique for each geometry, containing information about the lattice periodicity and the magnetic length scale. Each value of $\alpha$ defines a system of coupled equations and an associated matrix of size $q \times q$ (square), or $2q \times 2q$ (honeycomb). Choosing a large prime number, such as $q = 1123$, yields a dense eigenvalue spectrum, as shown in Fig. 1 (for $k_y = 0$). These plots exhibit fascinating self-similar structure, as well as reflection symmetries with respect to zero energy and half flux quantum.

## Antisymmetric Butterfly

Electrons in a triangular lattice in the presence of **B** were studied by F. H. Claro and G. H. Wannier.[4] Similar to the square lattice system, the vector potential $\mathbf{A} = (0, Bx, 0)$ generates magnetic phases along diagonal hoppings in the triangular geometry.[5] Using these phases and translation symmetry along $y$, the tight-binding equation for the triangular lattice is

$$\psi(m-2) + u_{m-1}\psi(m-1) + u_m\psi(m+1) + \psi(m+2) = \varepsilon\psi(m), \tag{4}$$

with coefficients

$$u_m = 2\cos\Big(\frac{\sqrt{3}}{2}k_y - 2\pi\alpha(m+\frac{1}{2})\Big), \tag{5}$$

The index $m$ labels sites in the magnetic supercell following the triangular geometry in Fig. 1c. The magnetic flux is $\Phi = BS$, with $S = \sqrt{3}a^2/2$ twice the area of a triangular plaquette of side $a$. In contrast to the energy spectrum of the symmetric butterflies, the energy spectrum of the triangular lattice has a period of 2 in $\alpha$. For the triangular butterfly, Eq. 3 of the main text can be transformed to $\Phi/\Phi_0 = 2\alpha$ because of the two triangular plaquettes inside the unit cell of the lattice, as schematically shown in Fig. 1c of the main text. This double plaquette leads to the double period in $\alpha$ for the antysymmetric butterfly seen in Fig. 2,[4] and the symmetry is recovered about $\alpha = 1$ instead of $\alpha = 1/2$ as in the

symmetric butterflies. The period of Eq. 4 is implicit in the coefficient $u_m = u_{m+q}$, and the wave functions fulfill $\psi(m) = \psi(m + q)$. The matrix representation for Eq. 4 has size $q \times q$, and yields a non-symmetric energy spectrum that however shows rich self-similar structure, as seen in Fig. 2.

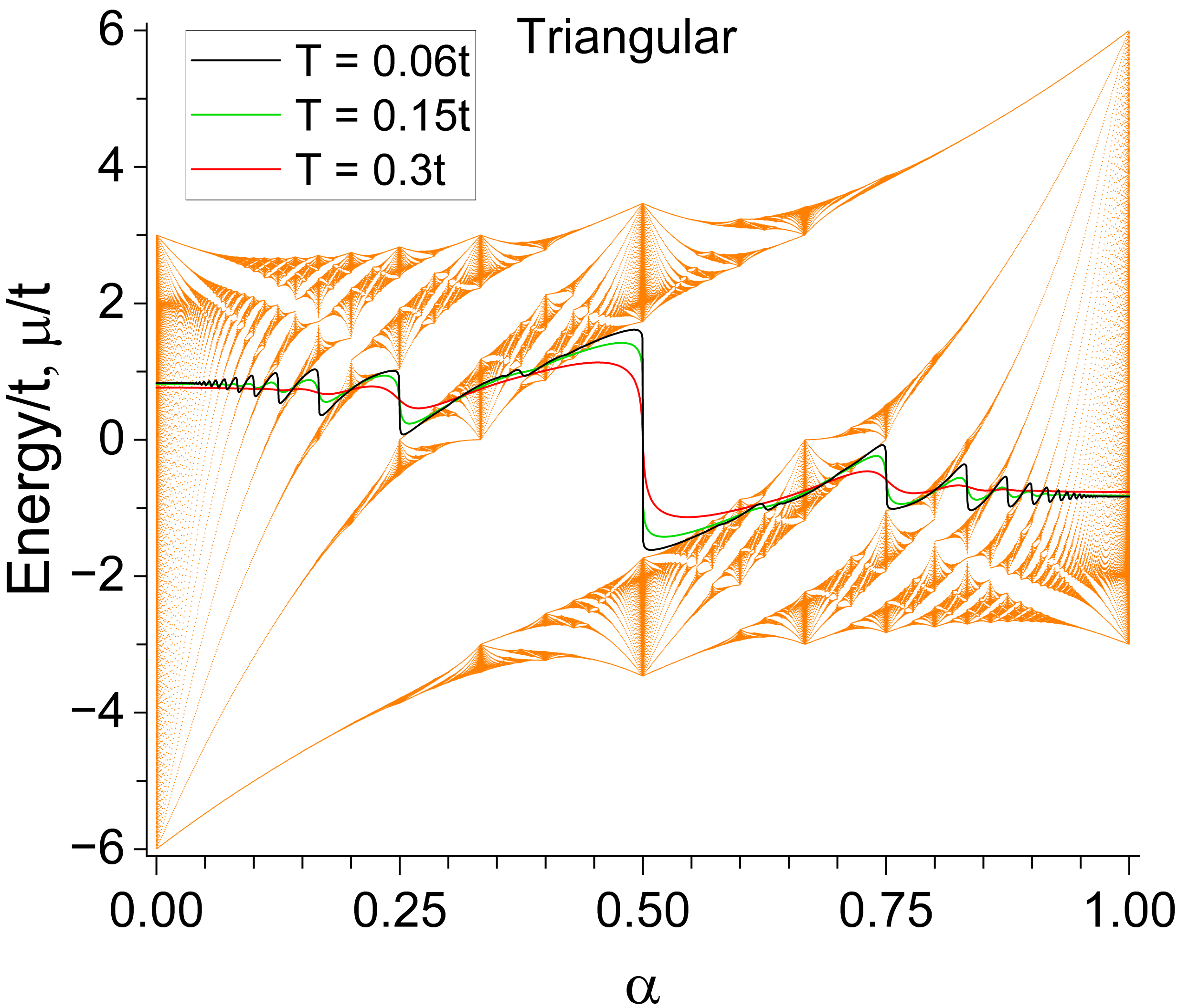


Figure 2: Chemical potential curves $\mu(\alpha, T)$ (solid lines) as a function of $\alpha = p/q$ ($q = 1123$) and different values of temperature $T$ under the condition of half-filling in the triangular lattice; all energies in units of the hopping parameter $t$. The energy spectrum $\varepsilon$ is shown behind the curves, where the sharp drops in $\mu$ are seen to occur within the gaps in the spectrum.

## Density of States

Numerical diagonalization of the matrices associated with Eqs. 1, 2, and 4, yields the eigenenergies $\{\varepsilon_l(\alpha)\}$, where the index $l$ runs from 1 to $Q$ for each value of $\alpha$, with $Q = q$ for the square and triangular lattice, and $Q = 2q$ for the honeycomb lattice. The density of states (DOS) is given by

$$D_\alpha(\varepsilon) = \sum_{l=1}^{Q} \delta\big(\varepsilon - \varepsilon_l(\alpha)\big). \tag{6}$$

A crucial characteristic of the square and honeycomb lattice spectra is that the reflection symmetry about $\varepsilon = 0$ ensures perfect electron-hole symmetry in the DOS, $D_\alpha(\varepsilon) = D_\alpha(-\varepsilon)$. On the contrary, $D_\alpha(\varepsilon) \neq D_\alpha(-\varepsilon)$ for the triangular lattice. We should note, however, that the full spectrum is traceless, so that $\sum_{l=1}^{Q} \varepsilon_l = 0$ for each $\alpha$ value in all three lattices. As we show below, these symmetry properties of the DOS are useful for setting the chemical potential $\mu$ in the systems.

### Thermodynamic quantities – symmetric butterflies

The electronic specific heat is calculated through its elementary definition $C_e = \partial U_e/\partial T$, with $U_e$ the electronic internal energy

$$U_e(\alpha, T) = \int \varepsilon D_\alpha(\varepsilon) f(\varepsilon,\mu, T) d\varepsilon = \sum_{l=1}^{Q} \varepsilon_l(\alpha) f(\varepsilon_l(\alpha), \mu, T), \tag{7}$$

where we have made use of Eq. 6. Using the established condition of half filling yielding $\mu = 0$, the specific heat has the form

$$C_e(\alpha, T) = \sum_{l=1}^{Q} \varepsilon_l(\alpha) \frac{\partial f_0}{\partial T} = \sum_{l=1}^{Q} \frac{\varepsilon_l^2(\alpha)}{4T^2 \cosh^2\left(\frac{\varepsilon_l(\alpha)}{2T}\right)}, \tag{8}$$

with $f_0 \equiv f(\varepsilon_l(\alpha), \mu = 0, T)$, and $k_B = 1$. The electronic entropy $S_e(\alpha, T)$ is given by

$$S_e(\alpha, T) = -\int D_\alpha(\varepsilon)[f \ln f + (1 - f) \ln(1 - f)] d\varepsilon, \tag{9}$$

in terms of the Fermi-Dirac function $f$. With the DOS in Eq. 6, and the condition $\mu = 0$, $S_e$ can be written as

$$S_e(\alpha, T) = \sum_{l=1}^{Q} \left[ \ln\left(1 + e^{-\varepsilon_l(\alpha)/T}\right) + \frac{\varepsilon_l(\alpha)}{T} f_0 \right]. \tag{10}$$

This expression avoids numerical instabilities that can arise from computing logarithms of very small numbers at low temperatures.

### Thermodynamic quantities – antisymmetric butterfly

The calculation of the electronic specific heat and entropy for the triangular lattice follows similar steps to those in the symmetric butterflies, here including the corresponding $\mu = \mu(\alpha, T)$ at half-filling. The internal energy is given by Eq. 7. The related specific heat is $C_e = \partial U_e / \partial T$. We find it convenient to directly compute the specific heat from the numerical derivative of $U_e$, using a small temperature step, $h = 0.001t$.

The electronic entropy for the triangular butterfly is correspondingly given by Eq. 10, with $\varepsilon_l$ replaced by $\varepsilon_l - \mu$, accordingly.

### Shape of the 'hearts'

It is interesting to analyze the shapes of the hearts on the square and honeycomb lattices in connection with their respective butterfly energy spectrum. In Fig. 3, superimposed plots of the specific heat in Fig. 3a and 4a of main text and the energy spectrum in Fig. 1 are shown to easily visualize how the shape of the hearts follows the $\alpha$-dependent spectral characteristics of each butterfly. For both lattices, each heart (brown zones) lies inside the energy gaps (the wings) of the spectrum, indicating $C_e$ is suppressed for the hearts up to moderate $T$.

The (a)symmetry axis of the hearts at different alpha matches the spines of the central and butterfly replicas. As described in the main text, the spines in the square lattice lie at

$\alpha = 1/2n$ (panel a), and $\alpha = 1/n$ (with $n$ integer) for the honeycomb lattice (panel b).

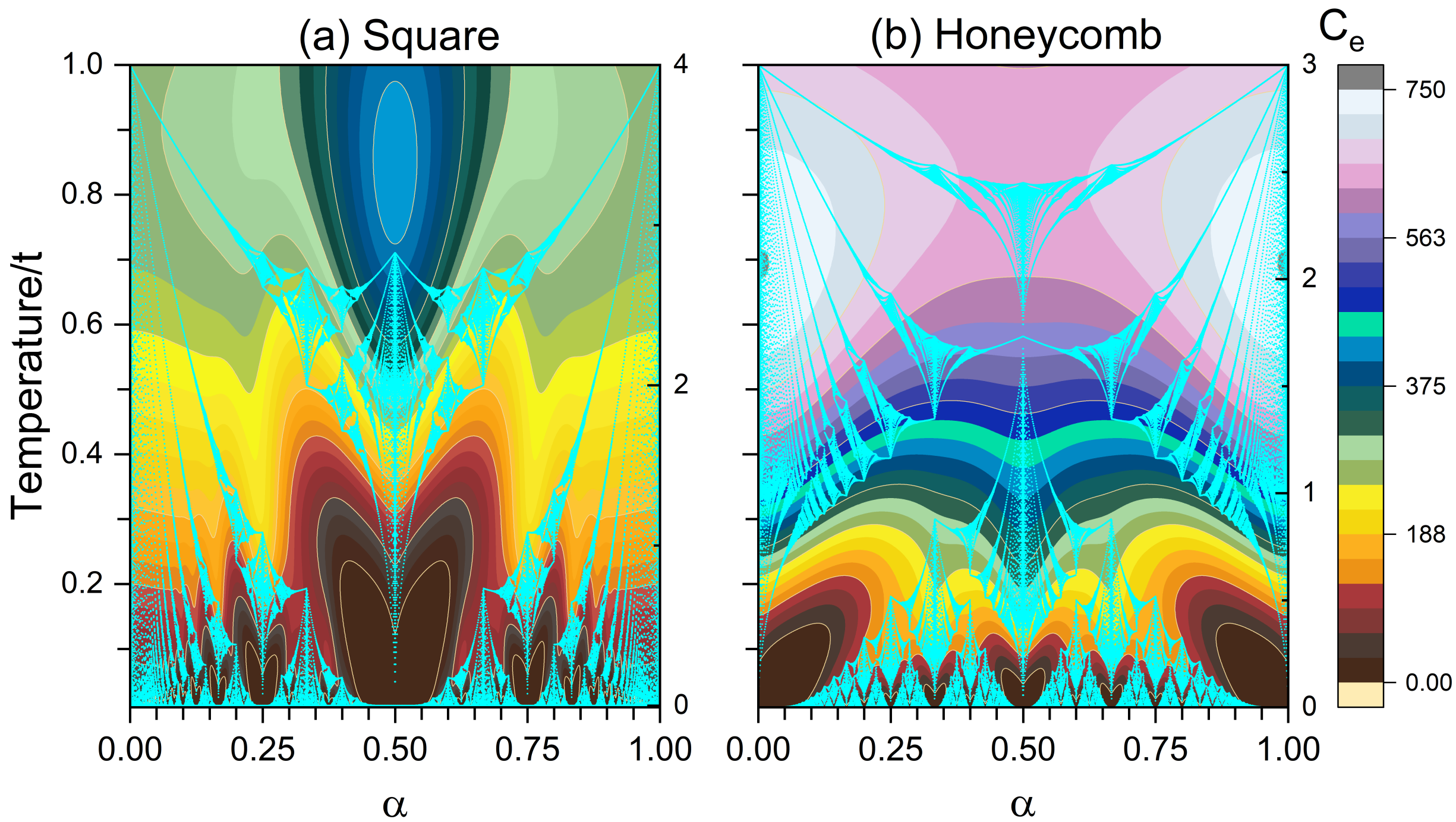


Figure 3: Contour plots for the specific heat $C_e$ as a function of temperature $T$ and $\alpha = p/q$ ($q = 1123$) for the square (a) and honeycomb (b) lattice. The spectrum in cyan color is superimposed on the $C_e$ plots, with the right vertical axes corresponding to its energy, here from $\varepsilon = 0$ to the positive maximum for each case. Notice that these contour plots are the same results as in Figs. 3a and 4a of main text.

The central hearts at $\alpha = 0.5$ in both lattices are the largest and most symmetric due to the largest and symmetric gaps in each spectrum about $\alpha = 0.5$. The smaller hearts at different $\alpha$ also follow the spectral gaps, but there are no more symmetric hearts due to the asymmetric energy gaps across those $\alpha$ values. A noticeable difference between both lattices happens near extreme $\alpha$, while the square butterfly presents many available quantized Landau levels at half filling near $\alpha = 0$ and $\alpha = 1$, the honeycomb butterfly presents a large spectral gap between consecutive Landau levels near those $\alpha$ regions. In those flux $\alpha$ regions, only half-hearts are preserved with low specific heat because of that single spectral butterfly wing gap.

For visual effects, a different view of the specific heat contours for each lattice geometry is shown in Fig. 4. This view allows a more linear visualization for $C_e$, thereby enabling a

clearer comparison among the three cases.

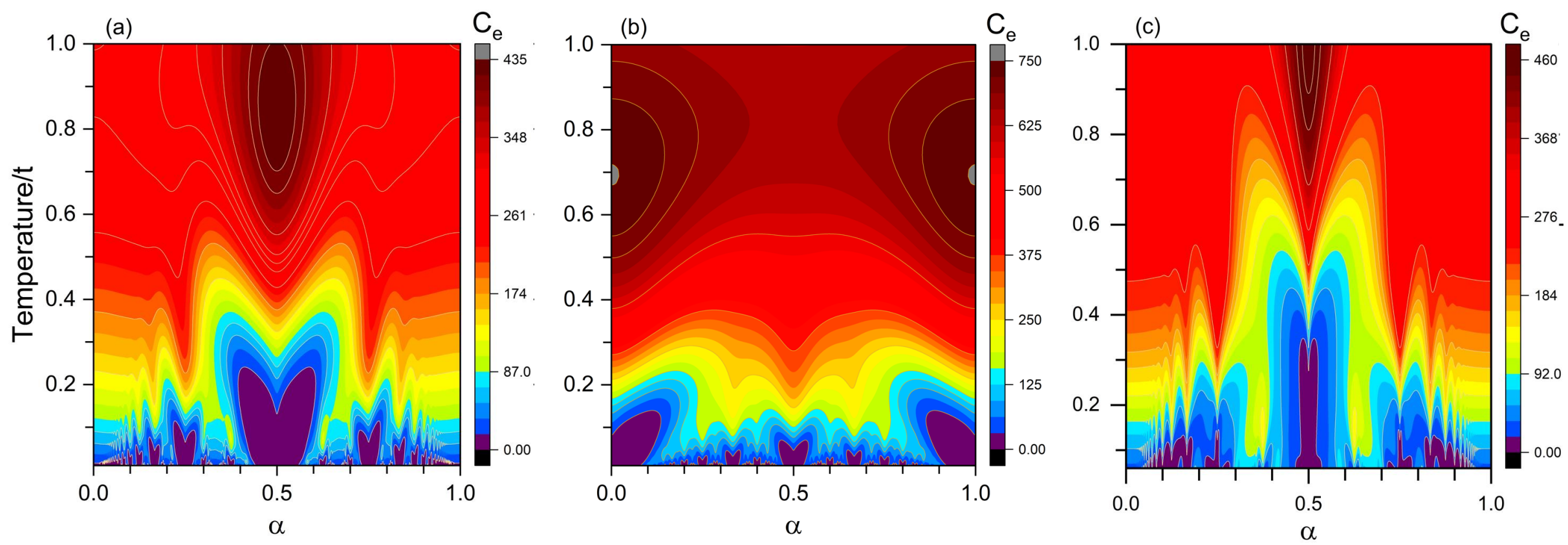


Figure 4: Countour plots for the specific heat $C_e$ as a function of temperature $T/t$ and $\alpha = p/q$ ($q = 1123$) for the square (a), honeycomb (b), and triangular (c) lattice. Notice that these contour plots are the same results as in Figs. 3a, 4a and 5a of main text but with different colors.

**Specific heat and entropy as a function of temperature**

To better understand the thermal behavior of the symmetry axis of the hearts (the central spine), and particular regions of the specific heat and entropy contours, we examine the temperature dependence of $C_e(T)$ and $S_e(T)$ at different $\alpha$ fluxes for the three lattices, Figs. 6 and 7. These curves allow one to relate the thermal energy of electrons, $k_BT$, to their subband structure in the corresponding butterfly energy spectrum.

**Square and Honeycomb lattices.** For the central hearts with symmetry axis at $\alpha = 0.5$ (the main spine of the butterflies) in the square and honeycomb lattices, $C_e$ remains suppressed at low temperatures because of the spectral characteristics with low DOS at half filling. The suppression is larger in $T$ for the square lattice, corresponding to a larger spine at $\alpha = 0.5$. After the suppression, the behavior of $C_e$ as $T$ increases shows local maxima in the honeycomb lattice due to its gapped nature in the spectrum at $\alpha = 0.5$. At this flux value, the thermal energy of electrons reaches the energy scale of the gaps, and they can be thermally activated across successive gaps.

The half of the hearts with symmetry axis at extreme $\alpha = 0$ and $\alpha = 1$ in the honeycomb lattice show a suppression in $C_e$ at low $T$, and this is in contrast to the square lattice, where $C_e$ starts to grow at very low $T$, see blue lines in Fig 6 top and central panels. This effect is directly connected to the thermal activation processes at those extreme flux values. Electrons at half filling have sufficient thermal energy $k_B T$ to activate at low $T$ due to tiny energy steps between successive subbands in the square lattice, of the order of $\Delta\varepsilon \approx 10^{-3}t$ for $q = 1123$. However, in the honeycomb energy spectrum, the subbands asymptotically approach each other at extreme $\alpha$, suppressing $C_e$ at low $T$, and electrons begin to activate as they overcome that energy scale.

There is another particularly interesting flux value to consider, namely $\alpha = 0.2$. At this flux, the $C_e$ contour of the square lattice intersects the edge of a heart-shaped replica, whereas in the honeycomb lattice it crosses a butterfly spine (an axis of asymmetry of one heart replica (see Fig. 1b). For both lattices, the DOS exhibits a pocket-like structure around zero energy, with intercalated gaps. This structure is more intricate in the honeycomb case, where additional peaks are observed (Fig. 5). These low-energy pockets in the DOS give rise to local maxima at low temperature in the electronic specific heat $C_e(T)$, as shown by the orange curves in the top and central panels of Fig. 6. As the temperature increases, electrons are thermally excited across the first energy gap, leading to a second peak in the specific heat. This feature appears at nearly the same temperature in both lattices, reflecting the comparable magnitude of the gap. However, differences in the higher-energy DOS structure result in distinct behavior of $C_e(T)$ at elevated temperatures.

The $S_e(T)$ curves displayed in Fig. 7 for the square lattice at $\alpha = 0.5$, show that the vanishing DOS near half filling results in a significantly slower entropy growth compared to $\alpha = 0$ and $\alpha = 0.2$ cases. This explains why the entropy minimum in the $T - \alpha$ map at flux $\alpha = 1/2$ (the larger tunnel in Fig. 3b of main text) coincide with the central butterfly spine: the restricted phase space for thermal excitations effectively mimics a gapped system at low temperature.

The entropy as a function of $T$ in the honeycomb lattice is notoriously different at low $T$ for the different fluxes we choose. Similar to $C_e$, a local smooth maximum and minimum are seen at low $T$ for flux $\alpha = 0.2$. At this flux there are multi-peaks and gaps and a butterfly spine near half filling producing those extreme values in $S_e(T)$. As $T$ increases, all $S_e(T)$ curves approach each other, electrons at high temperature overcome all the energy gap scales of the honeycomb spectrum, converging the entropy to almost the same value regardless of the flux.

**Triangular Lattice.** The specific heat $C_e(T)$ for the triangular lattice in Fig. 6 bottom panel shows similar behavior as for the square lattice at $\alpha = 0$, increasing from very low $T$ and stabilizing from medium $T \approx 0.5t$. This similarity is associated with the continuous subband structure at $\alpha = 0$ in both lattices, where electrons have enough $k_B T$ to overcome consecutive energy levels in the spectrum.

At $\alpha = 0.2$, a local minimum in $C_e(T)$ appears at low temperatures. For this flux, the chemical potential is $\mu \sim 0.8t$, placing it near the center of the first energy pocket in the DOS, where the density of states is finite but decreases with increasing energy. This leads to a reduction of the specific heat as the temperature rises in this regime. Moreover, for the second energy pocket to contribute to $C_e(T)$ electrons must first be thermally excited across the intervening gap. This additional activation process further suppresses the specific heat at low temperatures, giving rise to the observed minimum.

The case of flux $\alpha = 0.5$ in the triangular lattice shows a much sharper transition and persistence of its structure for the central heart as temperature increases. This effect is different when compared to the square and honeycomb lattice in $C_e$ contour maps (Fig. 5a main text and Fig. 4 above). In particular, for the specific heat as a function of $T$ in the triangular lattice, $C_e(T)$ is strongly suppressed up to $T \approx 0.25t$ because of the main butterfly gap, Fig. 6, then as $T$ increases, $k_B T$ electrons reach the energy scale of the main butterfly gap ($\approx 4t$), and $C_e(T)$ grows smoothly. Similarly, $S_e$ in Fig. 7 at $\alpha = 0.5$ shows

a large suppression up to about $T \approx 0.3t$, in contrast to other flux values. These vanishing specific heat and entropy coming from the main energy gap scale are the responsible of the persistence of the structure in $C_e$ and $S_e$ contour maps, Fig. 5 of main text.

This behavior could be linked to the reduced symmetry of the triangular lattice relative to the square and honeycomb lattices, including the absence of spectral inversion symmetry about zero energy, and the flux dependence on the chemical potential, $\mu(\alpha, T)$. Consequently, the sharper features in thermodynamic quantities such as the specific heat and entropy can be understood as a direct manifestation of the lower symmetry, which gives rise to more irregular and compacted structures observed in the $C_e(\alpha, T)$ and $S_e(\alpha, T)$ contour maps.

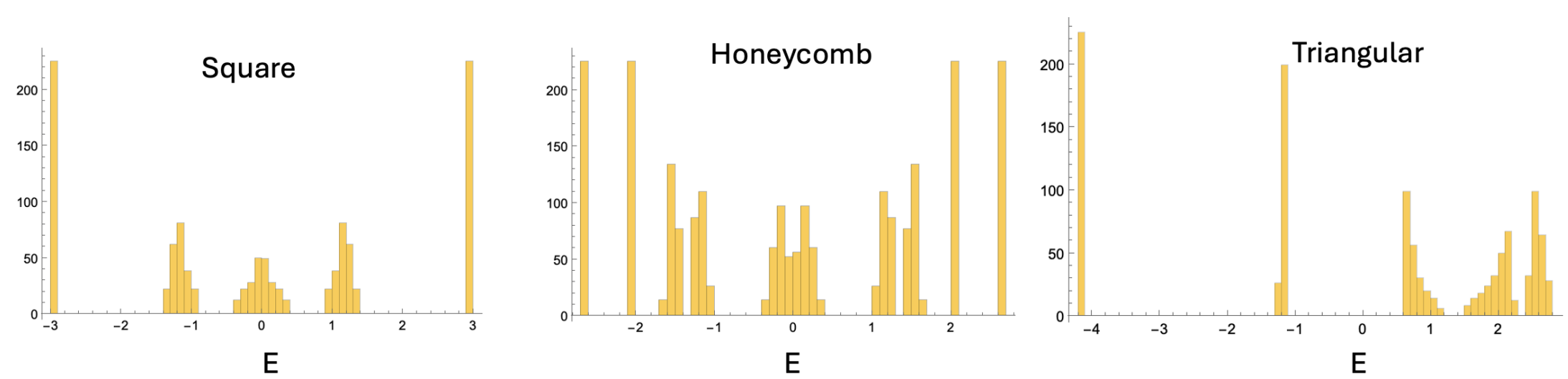


Figure 5: Density of states in arbitrary units as a function of energy $E/t$ at flux $\alpha = 0.2$, for the square, honeycomb, and triangular lattice, respectively.

**Different filling factor**

We have calculated a different filling factor away from half filling $\nu = 1/2$, namely $\nu = 0.6$, for the square lattice, as shown in Fig. 8. Here, deviations from half-filling ($\mu = 0$) make the chemical potential flux-dependent, breaking the spectral inversion symmetry about $\varepsilon = 0$, and rendering the chemical potential dependent on both flux and temperature, $\mu(\alpha, T)$, with direct consequences for thermodynamic quantities (not shown).

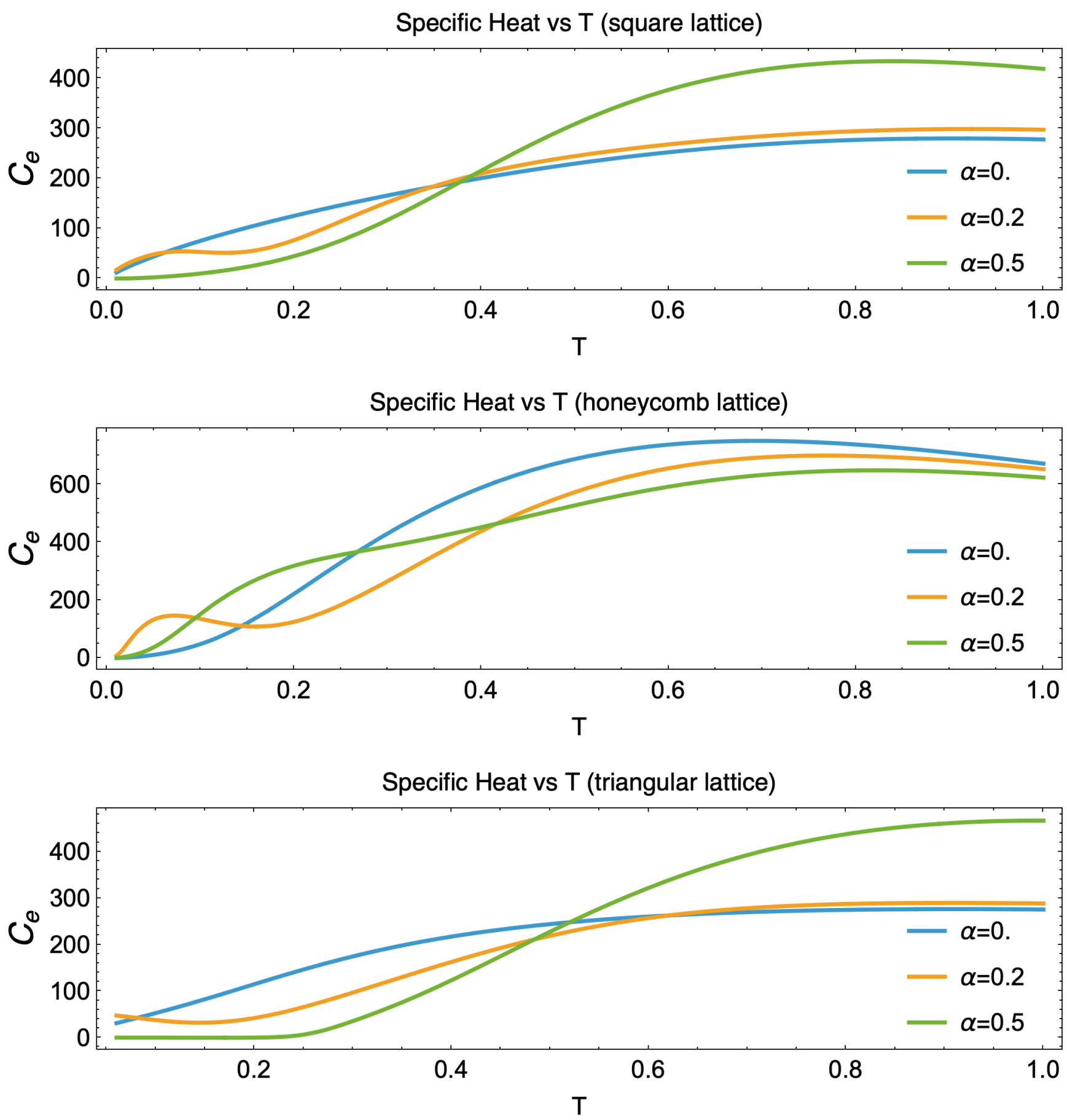


Figure 6: Specific heat as a function of temperature $T/t$ for different values of the flux, $\alpha = 0$, $\alpha = 0.2$, and $\alpha = 0.5$. Top, central, and bottom panels present the results for the square, honeycomb, and triangular lattice, respectively.

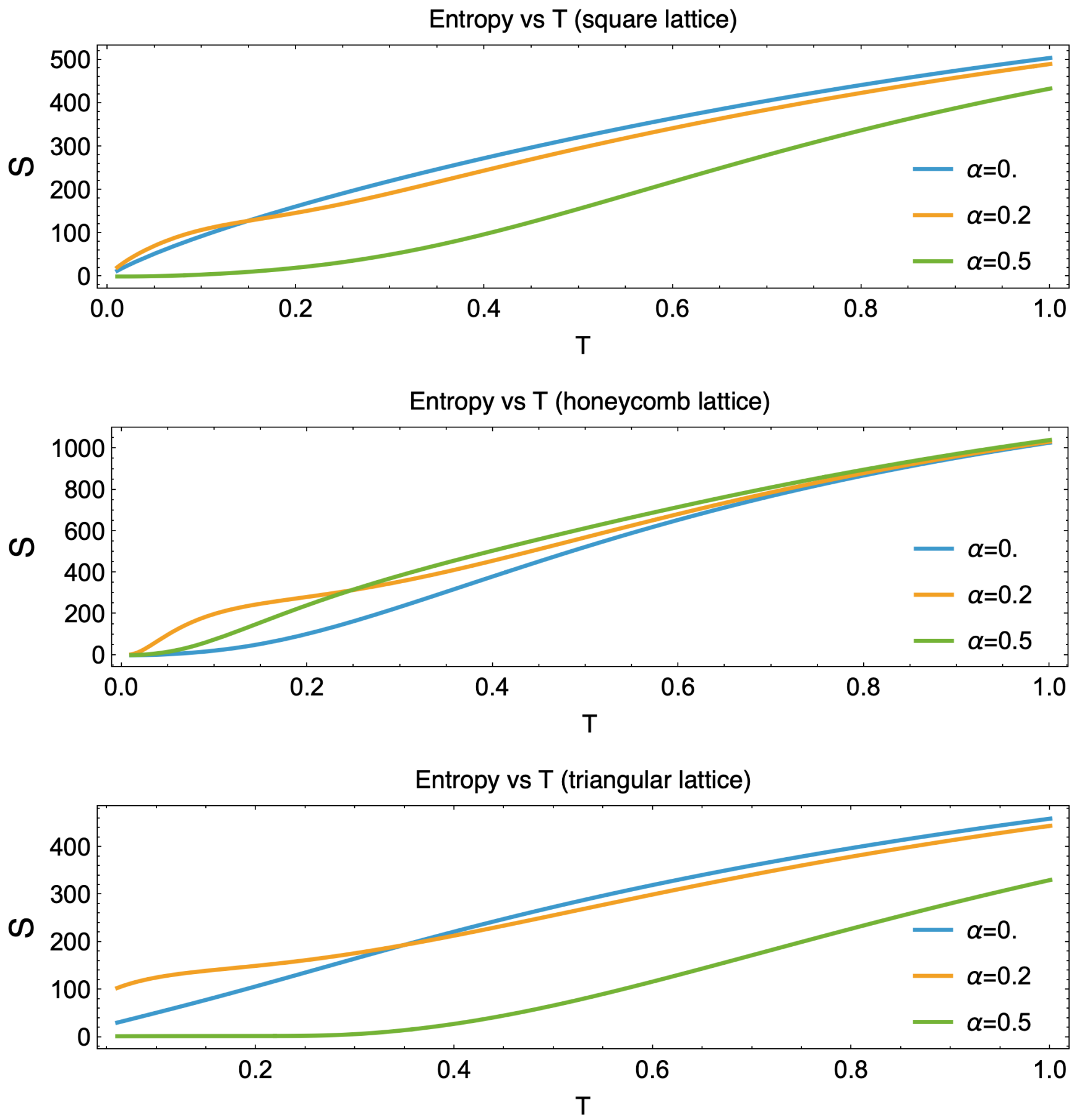


Figure 7: Entropy as a function of temperature $T/t$ for flux values $\alpha = 0$, 0.2, and 0.5. Top, central, and bottom panels present the results for the square, honeycomb, and triangular lattice, respectively.

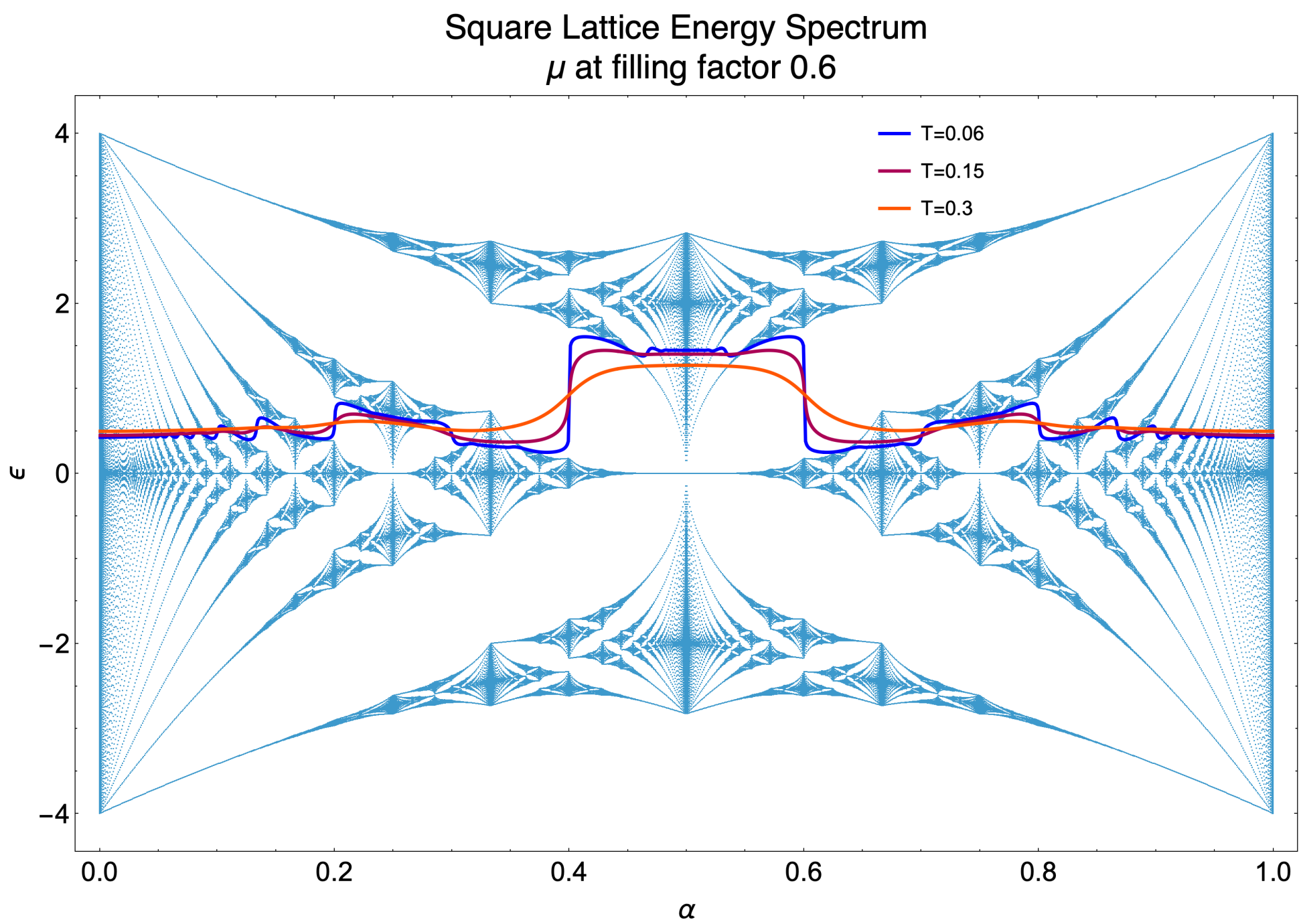


Figure 8: Flux and temperature-dependent chemical potential, $\mu(\alpha, T)$ at filling factor $\nu = 0.6$ in the square lattice for three temperatures $T = 0.06t$, $T = 0.15t$ and $T = 0.3t$.

### Experimental feasibility and MCE numbers

For realistic numbers in the magnetocaloric effect (MCE), we note that for moire graphene-based systems with lattice constants $a \approx 10$ nm, a flux $\alpha = 0.5$ corresponds to $B \approx 20$ Tesla. The temperature range $T/t \in [0, 1]$ corresponds to absolute temperatures up to $\sim 100$ K, using a hopping amplitude of $t = 10$ meV. Therefore we get immediate conversion from the dimensionless adiabatic temperature variation $\Delta(T/t)$ to an absolute adiabatic temperature change $\Delta T_{ad}$. Making the predicted magneto-thermo oscillations and MCE detectable in modern cryostat setups.

| dimensionless adiabatic temperature variation | adiabatic temperature change $\Delta T_{ad}$ (K) |
|---|---|
| $\Delta(T/t) = 0.001$ | 0.116 |
| $\Delta(T/t) = 0.005$ | 0.580 |
| $\Delta(T/t) = 0.010$ | 1.160 |
| $\Delta(T/t) = 0.020$ | 2.320 |

### About quantum capacitance

The quantum capacitance, also known as the chemical capacitance, is a fundamental concept in mesoscopic and nanoscale physics that arises whenever the density of electronic states of a system is finite and comparable to the geometrical capacitance scale. It was first introduced by Luryi[6] to account for the fact that, in low-dimensional or low-density-of-states systems, charging a capacitor requires not only the electrostatic energy to separate charge across the dielectric, but also the quantum kinetic energy needed to fill electronic states above the Fermi level.

The quantum capacitance $C_Q = e^2 \partial N/\partial\mu$, with $N$ the number of particles, can be written explicitly as

$$C_Q(\mu, T) = \frac{e^2}{4k_B T} \int_{-\infty}^{+\infty} D(E)\,\mathrm{sech}^2\!\left(\frac{E-\mu}{2k_B T}\right) dE. \qquad (11)$$

This expression has a transparent physical interpretation: $C_Q$ is a thermal convolution of the density of states with a bell-shaped $\mathrm{sech}^2$ kernel of width $\sim k_B T$, centered at the chemical potential $\mu$. At low temperatures, the kernel narrows toward a Dirac delta and one recovers $C_Q \to e^2\, D(\mu)$, so that the quantum capacitance directly measures the density of states at the chemical potential. At higher temperatures, the kernel broadens and $C_Q$ samples $D(E)$ over an increasingly wide energy window, washing out fine structure in the spectrum.

Using Eq. (11), we computed the quantum capacitance of the honeycomb lattice (graphene) as a function of the magnetic flux parameter $\alpha$ for different temperatures and compared it with the corresponding entropy. This comparison is natural because the weighting function appearing in the entropy expression, Eq. (9), closely resembles the $\mathrm{sech}^2$ kernel entering the quantum capacitance formula. The primary distinction is the prefactor $e^2/(4k_B T)$, which causes the quantum capacitance to decrease with increasing temperature, whereas the entropy always increases.

All calculations were performed at half-filling ($\mu = 0$), corresponding to the charge-neutrality (Dirac) point of the honeycomb lattice in the absence of a magnetic field. The

temperatures considered range from the low-temperature limit, $k_B T/t \ll 1$, where fine spectral details can be resolved, to the high-temperature regime, $k_B T/t \sim 1$, where thermal broadening significantly smooths the spectrum ($T/t \in [0.01, 1.00]$).

The results are presented below in Figs. 9, 10, and 11. All of these results indicate that quantum capacitance at low temperatures can also be seen as an experimentally accessible thermal quantity for resolving the fractal nature of the butterflies.

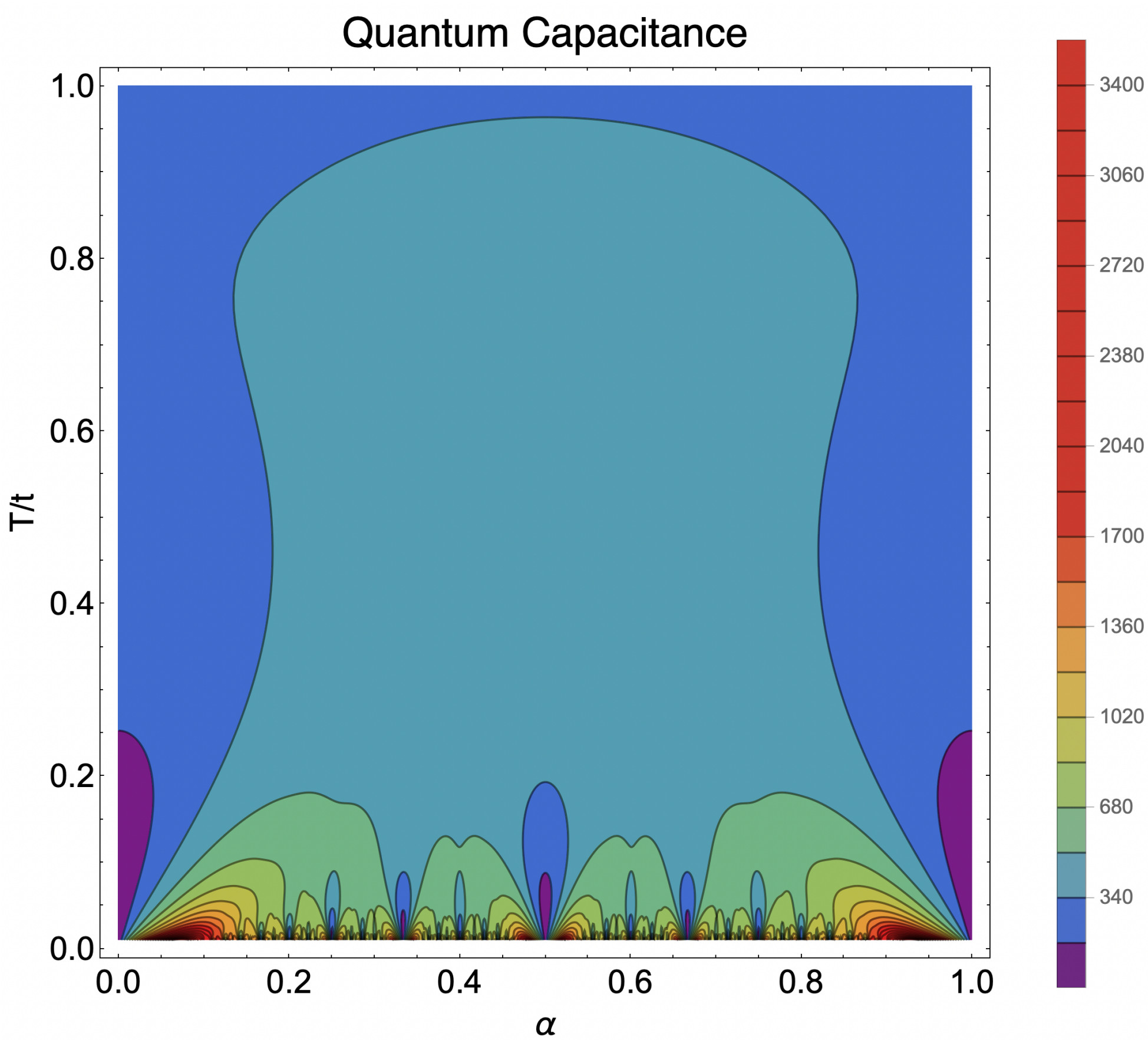


Figure 9: Contour plot for the quantum capacitance $C_Q$ as a function of temperature $T/t$ and $\alpha = p/q$ ($q = 1123$) for the honeycomb lattice. We observe that $C_Q$ decreases as temperature increases, and a bell shape is formed across $\alpha$ and $T/t$. At low temperatures, fine structure appears, where $C_Q$ shows maxima (red contours), and drop-like shapes centered at diverse $\alpha$ that coincide with the butterfly spines.

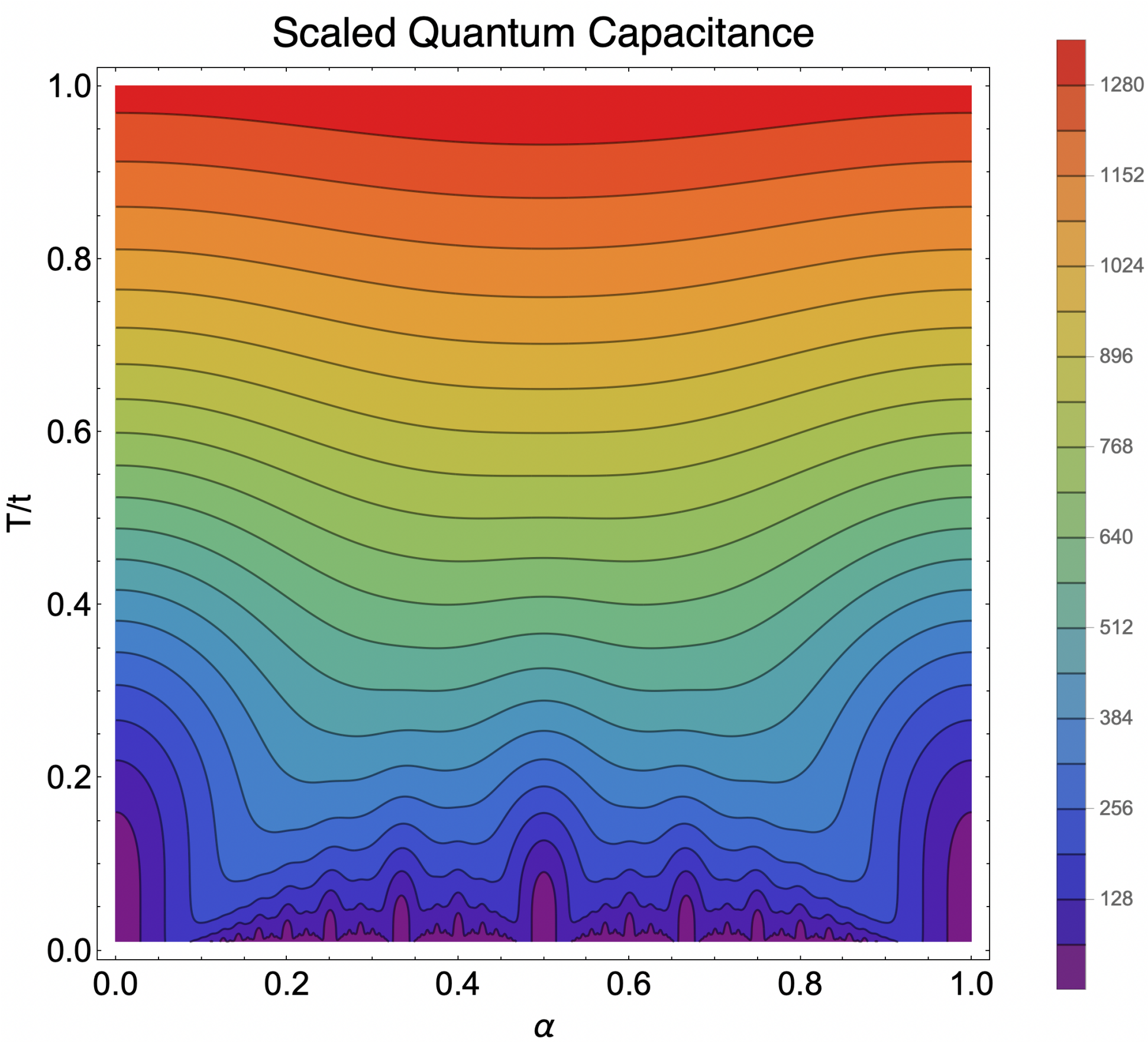


Figure 10: Contour plot for the scaled quantum capacitance, $C_Q \times 4k_BT/e^2$, as a function of temperature $T/t$ and $\alpha = p/q$ ($q = 1123$) for the honeycomb lattice. We find that the function closely tracks the entropy–see Fig. 4b in the main text.

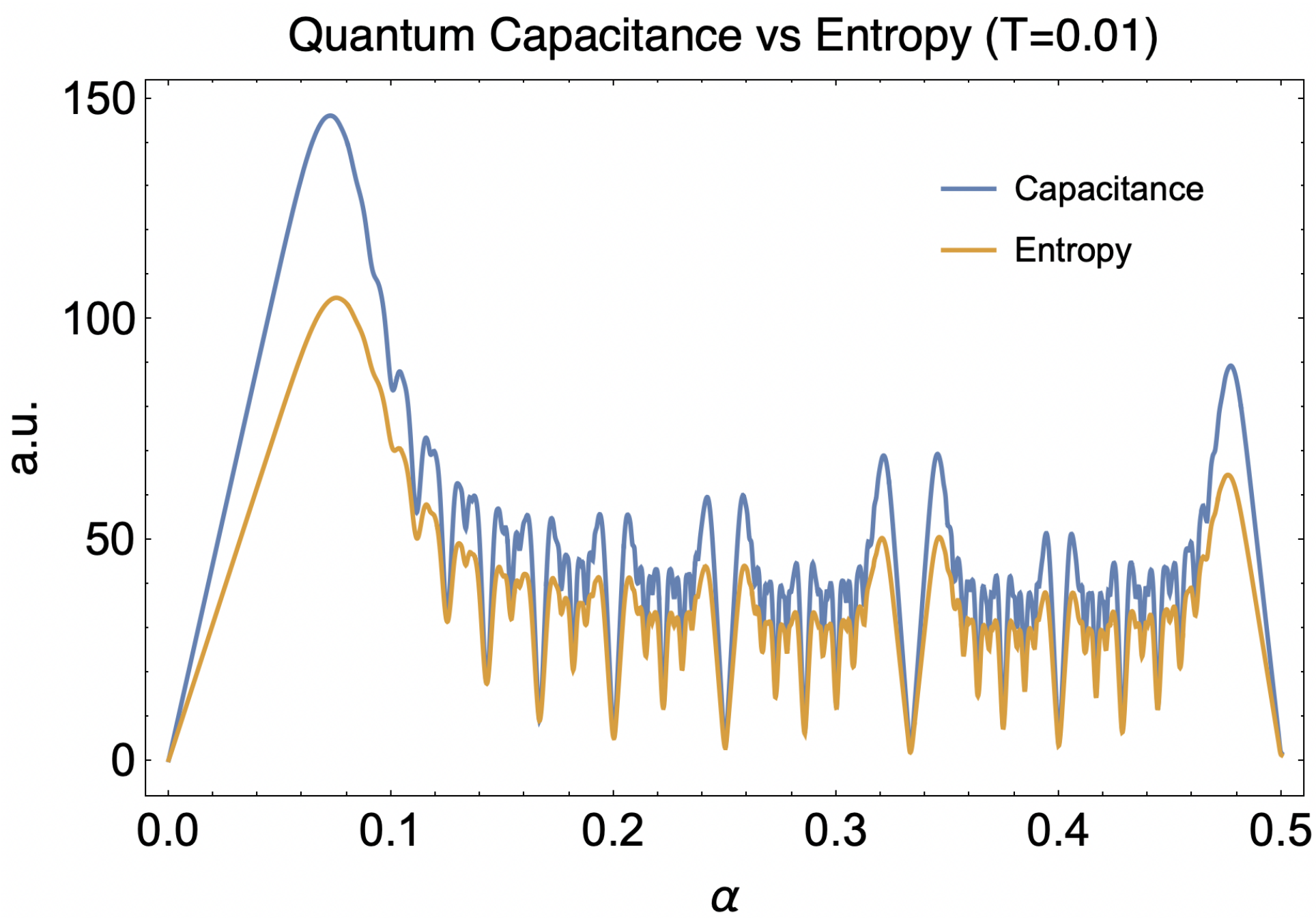


Figure 11: Quantum capacitance and entropy $S_e$ as a function of $\alpha = p/q$ ($q = 1123$) at $T = 0.01t$ for the honeycomb lattice. The function $C_Q$ at this temperature exhibits a remarkably similar line-shape behavior to that of the entropy, with a slightly increased magnitude across $\alpha$.

# References


(1) Hofstadter, D. R. Energy levels and wave functions of Bloch electrons in rational and irrational magnetic fields. *Phys. Rev. B* **1976**, *14*, 2239–2249.

(2) Satija, I. The Hofstadter Butterfly: Bridging Condensed Matter, Topology, and Number Theory. 2025; `https://arxiv.org/abs/2507.13418`.

(3) Canonico, L. M.; García, J. H.; Rappoport, T. G.; Ferreira, A.; Muniz, R. Shubnikov–de Haas oscillations in the anomalous Hall conductivity of Chern insulators. *Phys. Rev. B* **2018**, *98*, 085409.

(4) Claro, F.; Wannier, G. Magnetic subband structure of electrons in hexagonal lattices. *Phys. Rev. B* **1979**, *19*, 6068.

(5) Oh, G.-Y. Comment on “Hofstadter butterfly for the hexagonal lattice”. *Phys. Rev. B* **2001**, *63*, 087301.

(6) Luryi, S. Quantum capacitance devices. *Appl. Phys. Lett.* **1988**, *52*, 501–503.
